# Waste‐Derived Fine‐Milled Bone Char for Apparent Cr (VI) Removal From Water and Preliminary Asphalt‐Compatible Processing of the Recovered Residue

**DOI:** 10.1002/wer.70580

**Published:** 2026-09-17

**Authors:** Kevin Yonny Avila‐Robayo, Eduardo J. Rueda, Jaime Plazas‐Tuttle

**Affiliations:** ^1^ Department of Civil and Environmental Engineering Universidad de Los Andes Bogotá Colombia; ^2^ Escuela de Construcción Civil, Facultad de Ingeniería Pontificia Universidad Católica de Chile Santiago Chile; ^3^ Water Distribution and Sewerage Systems Research Center (CIACUA) Universidad de Los Andes Bogotá Colombia

**Keywords:** adsorption, asphalt‐compatible matrices, bone char, Cr (VI), industrial wastewater treatment, process residue, spent adsorbent management, tannery wastewater

## Abstract

Chromium adsorption transfers contamination to a solid residual that still requires management. This proof‐of‐concept study evaluated fine‐milled bone char (BC; D50 = 5.52 μm) for apparent aqueous Cr (VI) removal from acidic synthetic water and examined asphalt‐compatible processing of the recovered residue. Contact‐time screening showed > 97% apparent Cr (VI) removal within 30 min; 120 min was retained only as a standardized operational contact time because later archived residual concentrations were very low, the assay lacked independent replication, and the sample‐equivalent lower calibration bound could not be reconstructed from the available records. Observed apparent aqueous‐depletion‐based uptake reached 4.39 mg g^−1^ in one screening condition. Nonlinear Langmuir, Freundlich, Sips, and Redlich–Peterson models empirically described the refined 120‐min dose–response dataset, with Freundlich receiving the greatest Akaike information criterion (AICc) support. Drying the acidified BC/Cr system produced 22.65 g of unresolved additional dry material beyond the initial BC and nominal Cr input, most of which was water‐soluble upon rehydration. The resulting process‐derived residue was incorporated into asphalt binder at 3 and 6 wt.% without large descriptive shifts in binder or mortar master curves. An adapted nonstandard mortar‐cylinder cyclic recovery test showed higher mean recovery with increasing residue dosage, but these descriptive values are not directly comparable with standard binder MSCR results. Leaching, chromium speciation, residue characterization, and independent replication are required before reuse or containment claims.

## Summary

- Fine‐milled bone char produced rapid apparent aqueous Cr (VI) depletion under acidic synthetic‐water conditions.
- Removal efficiency and apparent uptake depended strongly on dose and initial Cr (VI) concentration.
- The dried process residue contained a large unresolved water‐soluble fraction.
- Asphalt incorporation produced no large descriptive shifts in binder or mortar master curves.
- Leaching and chromium speciation must be tested before reuse or containment claims.

## Graphical abstract

Waste‐derived ultrafine bone char removed Cr (VI) from acidic synthetic water, and the recovered Cr‐loaded solid was incorporated into asphalt‐compatible binder and mortar without major rheological disruption. The pathway links contaminant capture with preliminary spent adsorbent management, but leaching, chromium speciation, aging, and real‐wastewater validation remain required.

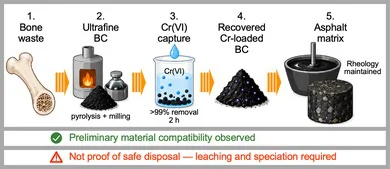

## 1 Introduction

Chromium‐bearing effluents from leather‐tanning operations remain a persistent water quality concern because chromium mobility, toxicity, and treatability depend strongly on oxidation state and wastewater chemistry (Bhardwaj et al. 2023; Burbridge et al. 2012; Zhao et al. 2022). Chromium (III) is widely associated with tanning processes and can form less mobile precipitates or complexes under specific pH and redox conditions, whereas hexavalent chromium (Cr (VI)) is generally more soluble, mobile, and toxic in aquatic systems (Burbridge et al. 2012; Costa and Klein 2006; Zhitkovich 2011). This distinction is especially important in tannery‐impacted waters, where pH, salts, sulfides, organic matter, suspended solids, and redox‐active constituents may alter chromium speciation, partitioning, and downstream exposure risk (Bhardwaj et al. 2023; Burbridge et al. 2012; Zhao et al. 2022). Therefore, treatment strategies for tannery‐related discharges must address not only aqueous chromium removal but also the residual fate of chromium after treatment.

Adsorption is frequently investigated for Cr (VI) removal because it is operationally simple, adaptable to decentralized or batch treatment configurations, and compatible with low‐cost or waste‐derived adsorbents (Hart et al. 2023; Kreetachat et al. 2026; Medellín Castillo et al. 2023; Rajapaksha et al. 2022). However, adsorption does not destroy chromium; it transfers the contaminant from the aqueous phase to a solid phase. High removal efficiency is therefore insufficient unless the contaminant‐loaded adsorbent is also recovered and managed to avoid secondary release (Baskar et al. 2022; Velempini et al. 2023). This residual‐management challenge is particularly relevant for Cr (VI)‐laden materials because improper handling can shift the environmental burden from contaminated water to a hazardous solid residual.

Bone char (BC) is a relevant waste‐derived adsorbent for chromium treatment because animal bone residues can be thermally converted into a mineral–carbon material containing calcium‐phosphate phases and carbonaceous domains (Hart et al. 2023; Medellín Castillo et al. 2023; Patel et al. 2015; Tansomros et al. 2024). Previous studies on BC and hydroxyapatite‐based materials have attributed metal uptake to combinations of ion exchange, surface complexation, precipitation‐related interactions, and binding with calcium‐ or phosphate‐associated sites (Avola et al. 2023; Flores‐Cano et al. 2016; C. Rojas‐Mayorga et al. 2016). However, the relative contribution of these pathways depends on precursor origin, thermal conversion conditions, mineral composition, porosity, particle size, surface chemistry, pH, competing ions, and chromium speciation (Hart et al. 2023; Medellín Castillo et al. 2023; C. K. Rojas‐Mayorga et al. 2013). Therefore, this study interprets Cr (VI) removal at the level directly supported by batch adsorption tests and does not assign a dominant removal mechanism without additional chromium‐speciation or post‐adsorption surface characterization.

Functionalized or composite bone char adsorbents may achieve higher Cr (VI) uptake than unmodified materials, but they also require additional reagents, synthesis steps, activation procedures, or material complexity (Avola et al. 2023; de Melo et al. 2018; Ranjbar et al. 2018). In contrast, the present study intentionally used a low‐complexity, fine‐milled BC fraction produced by thermal treatment and mechanical size conditioning. Particle‐size reduction was used to improve dispersion and potential surface accessibility, but it should not be interpreted as evidence of a nanoscale material or as proof of a specific adsorption mechanism. The value of this approach is not to maximize adsorption capacity at any cost but to evaluate whether a simple waste‐derived adsorbent can be coupled with a posttreatment residual‐management pathway.

A critical limitation of many adsorption studies is that contaminant removal and posttreatment residual fate are often considered separately. Removal percentages, contact‐time trends, and isotherm‐model descriptions characterize aqueous treatment performance, but they do not establish what happens to the contaminant‐bearing solid after separation (Baskar et al. 2022; Velempini et al. 2023). For metal‐bearing spent adsorbents, postadsorption management strategies include regeneration, thermal or chemical stabilization, ceramic incorporation, cementitious solidification/stabilization, and geopolymer‐based matrices (Baskar et al. 2022; Chen et al. 2009; Raj et al. 2022; Tian et al. 2022; Verbinnen et al. 2015; Zein et al. 2022). Regeneration can reduce fresh‐adsorbent demand, but it may generate a metal‐enriched regenerant and requires evidence of retained adsorption performance across repeated adsorption–desorption cycles (Baskar et al. 2022; Yu and Yang 2024). Construction‐matrix incorporation is likewise a hypothesis requiring direct leaching and durability validation, not evidence of safe disposal (Raj et al. 2022; Wang and Wang 2022; Zein et al. 2022).

Asphalt‐compatible matrices are relevant for a preliminary incorporation assessment because asphalt binders, mastics, mortars, and mixtures routinely include mineral fillers, fines, bio‐based additives, carbonaceous materials, and engineered modifiers to adjust rheological or mechanical response (Bhat and Mir 2021; Huang et al. 2024; Musco et al. 2024; Yaro et al. 2023). From this materials perspective, the process‐derived bone char residue can be evaluated as a filler‐like modifier or binder‐extension candidate. However, pavement materials may experience stormwater contact, wet–dry cycling, aging, cracking, abrasion, runoff exposure, and end‐of‐life recycling, all of which may influence contaminant release (Kriech and Osborn 2022; Legret et al. 2005; Spreadbury et al. 2021). Asphalt compatibility therefore cannot be interpreted as chromium immobilization or safe disposal; it only establishes whether the recovered process residue can be incorporated without large adverse changes in binder or mortar response under the tested laboratory conditions.

Despite extensive research on Cr (VI) adsorption and growing interest in waste‐derived adsorbents, comparatively little experimental work connects aqueous metal removal with characterization and subsequent construction‐material processing of the recovered treatment residual (Raj et al. 2022; Zein et al. 2022). This gap is important because an adsorbent that performs well in water may still create a secondary residual‐management challenge after separation. Conversely, asphalt‐modification studies typically focus on rheological or mechanical performance and rarely consider modifiers derived from contaminant‐exposed water‐treatment residuals. The novelty of the present work is therefore the experimentally bounded linkage of aqueous Cr (VI) removal, recovered residue mass balance, and asphalt‐compatible processing, while explicitly leaving environmental containment as an unresolved validation requirement.

Accordingly, this study evaluates a preliminary capture‐to‐asphalt‐compatible processing pathway using fine‐milled BC produced from bovine bone waste. The specific objectives were to (i) prepare and size‐condition a fine‐milled bc adsorbent; (ii) evaluate apparent aqueous Cr (VI) removal under controlled synthetic‐water conditions; (iii) describe the refined 120‐min dose–response dataset using nonlinear Langmuir, Freundlich, Sips, and Redlich–Peterson isotherm models; and (iv) assess whether the process‐derived residue recovered after acidic Cr (VI) exposure and drying can be incorporated into asphalt binder and asphalt mortar without large descriptive changes in penetration or rheological response. The study is framed as a laboratory proof‐of‐concept for material compatibility, not as a demonstration of safe disposal, permanent chromium immobilization, or field‐ready containment. Leaching, chromium speciation, residue characterization, long‐term aging, abrasion, runoff exposure, regeneration, and real tannery wastewater testing remain outside the present scope and are required before environmental implementation.

## 2 Materials and Methods

### 2.1 Materials, Reagents, and Equipment

Bovine femur bone was used as the precursor for BC production. The bone was obtained from a slaughterhouse near Bogotá, Colombia, and processed through cleaning, drying, thermal treatment, mechanical milling, and particle‐size conditioning before adsorption and asphalt‐compatible incorporation experiments. A virgin 60–70 penetration‐grade asphalt binder supplied by the Civil Engineering Laboratory of Universidad de Los Andes was used for binder modification and asphalt mortar preparation.

Synthetic Cr (VI) solutions were prepared from potassium dichromate (K_2_Cr_2_O₇, Sigma‐Aldrich/Merck, ACS reagent grade, ≥ 99.0%). Sulfuric acid (H_2_SO₄, Sigma‐Aldrich/Merck, ACS reagent grade, 95.0%–98.0%) was used for acidification and pH adjustment. Dissolved Cr (VI) was quantified using the 1,5‐diphenylcarbazide colorimetric method. Distilled water was used for solution preparation, dilution, washing, and BC suspension preparation. A detailed list of reagents, equipment, and operating conditions is provided in Section S1 and Table S1.1.

### 2.2 BC Preparation and Particle‐Size Conditioning

BC was prepared from cleaned and dried bovine femur bone by thermal treatment under a nitrogen atmosphere. Approximately 100 g of crushed bone was distributed in ceramic crucibles, heated using a 5 h temperature ramp, and maintained between 700°C and 800°C for 2 h. The resulting bone‐derived char was cooled inside the furnace and recovered for subsequent mechanical size conditioning. No chemical activation or surface functionalization was applied before adsorption experiments.

The recovered BC was processed through sequential mechanical milling to obtain a finer working fraction. The first milling stage generated the BC‐MA fraction, whereas additional planetary ball milling generated the finer BC‐MB fraction. BC‐MB was selected for adsorption and asphalt‐compatible incorporation because it showed the smaller particle‐size distribution after sonication‐assisted dispersion. Detailed preparation steps, recovered masses, and milling‐support data are provided in Section S2 and Tables S2.1–S2.3.

### 2.3 Particle‐Size Analysis

Particle‐size distributions of BC‐MA and BC‐MB were determined by laser diffraction after sonication‐assisted aqueous dispersion. Characteristic particle‐size percentiles were reported as D10, D50, and D90, corresponding to the particle diameters below which 10%, 50%, and 90% of the measured distribution are found, respectively. The mean particle diameter was also reported.

The selected BC‐MB fraction is described as fine‐milled BC with a submicron fraction because its laser diffraction distribution had D10 = 0.78 μm, while D50 = 5.52 μm and D90 = 23.32 μm remained in the micrometer range. The material is therefore not described as nanoscale or nanoparticulate. Additional milling and dispersion details are provided in Section S2, especially Tables S2.2 and S2.3.

### 2.4 Cr (VI) Quantification

Dissolved Cr (VI) was quantified by ultraviolet–visible (UV–Vis) spectrophotometry using the 1,5‐diphenylcarbazide colorimetric method. Under acidic conditions, Cr (VI) reacts with 1,5‐diphenylcarbazide to form a colored complex measured at 540 nm. Calibration standards covered 0.00–0.40 mg L^−1^ Cr (VI), with 0.020 mg L^−1^ as the lowest nonzero standard in the analytical flask. For the dose–response assays, archived experimental records document that samples from the nominal 2 mg L^−1^ series were analyzed using a 10 mL of aliquot diluted to 50 mL (fivefold dilution), whereas samples from the nominal 20 mg L^−1^ series used a 1 mL of aliquot diluted to 50 mL (50‐fold dilution). Accordingly, the lowest nonzero calibration standard corresponds to sample‐equivalent concentrations of 0.10 and 1.0 mg L^−1^, respectively, before dilution. Concentrations were calculated from the calibration curve and corrected by the corresponding dilution factor. Reagent blanks and process blanks without BC were included as analytical and experimental controls, respectively. For the contact‐time assay, the archived procedure confirms dilution of the supernatant to a final volume of 50 mL but does not record the aliquot volume; therefore, a sample‐equivalent lower calibration bound cannot be reconstructed reliably for that assay. A formal method detection limit and quantification limit were not established from replicate blank/calibration statistics. Consequently, very low residual concentrations from the contact‐time assay and dose–response measurements falling below the corresponding sample‐equivalent calibration range are retained as archived measurements but are interpreted qualitatively rather than as quantitatively resolved concentrations. Calibration and dilution records are provided in Section S3.

### 2.5 Batch Adsorption Experiments

Batch adsorption experiments were conducted under controlled synthetic‐water conditions to evaluate apparent aqueous Cr (VI) removal by the selected fine‐milled BC fraction. The workflow included the following: (i) a contact‐time screening assay to select a standardized operational contact time and (ii) dose–response adsorption assays to evaluate removal efficiency and apparent uptake as a function of initial Cr (VI) concentration and BC dose. All tests were conducted in 15 mL polypropylene tubes using potassium‐dichromate solutions. BC suspensions were sonicated before use and maintained under magnetic stirring before dosing to improve suspension homogeneity.

The contact‐time screening assay was conducted at an initial Cr (VI) concentration of 2 mg L^−1^ and a BC dose of 1 g L^−1^. Single BC‐containing samples were analyzed after 30, 60, 120, 180, and 240 min, with a corresponding process blank without BC at each time. After contact, samples were centrifuged at 4000 rpm for 5 min, and the supernatant was analyzed for residual Cr (VI). Because the assay was designed to select a standardized contact time rather than estimate kinetic‐model parameters, 120 min was retained as the operational contact time for subsequent experiments; it is not treated as a statistically demonstrated equilibrium time.

Dose–response adsorption assays were then conducted at initial Cr (VI) concentrations of 2 and 20 mg L^−1^ using the standardized 120‐min operational contact time. An initial screening assay evaluated BC doses between 1 and 10 g L^−1^. A refined dose–response assay was subsequently performed to improve data resolution across the tested adsorbent‐to‐contaminant ratios. In the refined assay, BC doses of 0.1, 0.2, 0.5, 0.7, and 1.0 g L^−1^ were evaluated for 2 mg L^−1^ Cr (VI), whereas doses of 1, 2, 5, 7, and 10 g L^−1^ were evaluated for 20 mg L^−1^ Cr (VI). Each dose condition was represented by one independently prepared batch tube (*n* = 1), with a corresponding no‐BC blank for each initial Cr (VI) concentration. Accordingly, the dose–response dataset was interpreted descriptively and was not used for inferential statistical testing. Contact‐time sample preparation records and raw contact‐time adsorption results are provided in Section S4 and Tables S4.1–S4.2. Screening and refined dose–response sample preparation records and raw datasets are provided in Section S4 and Tables S4.3–S4.6.

Apparent aqueous Cr (VI) removal efficiency and apparent aqueous‐depletion‐based uptake were calculated using Equations (1) and (2) as follows:

(1) $$R\left(\%\right)=\frac{C_{0}-C_{e}}{C_{0}}\times 100$$

(2) $$q_{e}=\frac{V}{m}\left(C_{0}-C_{e}\right)$$

where R is the apparent aqueous Cr (VI) removal efficiency (%), $q_{e}$ is the apparent aqueous‐depletion‐based uptake (mg g^−1^), $C_{0}$ is the initial aqueous Cr (VI) concentration (mg L^−1^), $C_{e}$ is the residual aqueous Cr (VI) concentration measured at the standardized 120‐min endpoint (mg L^−1^), *V* is the solution volume (L), and *m* is the mass of bc added (g). Because only dissolved Cr (VI) was quantified, $q_{e}$ represents apparent uptake calculated from aqueous Cr (VI) depletion and does not by itself distinguish adsorption from possible redox or other phase‐transfer processes. Blank samples were used to identify non‐adsorptive concentration changes and were not included as adsorbent‐containing observations. Here and throughout the manuscript, the subscript e is retained for consistency with conventional isotherm notation; $C_{e}$ and $q_{e}$ refer to the measured concentration and calculated apparent uptake at the standardized 120‐min endpoint and do not imply that equilibrium was independently demonstrated.

### 2.6 Adsorption Isotherm Modeling

The refined 120‐min dose–response dataset was fitted directly in $q_{e}$‐versus‐$C_{e}$ space using nonlinear least squares. Four commonly used adsorption isotherm models were evaluated: Langmuir, Freundlich, Sips, and Redlich–Peterson. Because equilibrium was not independently demonstrated experimentally, these models are used here as empirical descriptors of the 120‐min dose–response dataset rather than as equilibrium design relationships or evidence of a specific adsorption mechanism. For consistency with conventional isotherm notation, the endpoint concentration and apparent uptake at the standardized 120‐min contact time are denoted $C_{e}$ and $q_{e}$, respectively; use of the subscript e does not imply that equilibrium was independently demonstrated.

The Langmuir model was expressed as Equation (3):

(3) $$q_{e}=\frac{q_{\max}K_{L}C_{e}}{1+K_{L}C_{e}}$$

The Freundlich model was expressed as Equation (4):

(4) $$q_{e}=K_{F}{C_{e}}^{1/n}$$

The Sips model was expressed as Equation (5):

(5) $$q_{e}=\frac{q_{S}{\left(K_{S}C_{e}\right)}^{n_{S}}}{1+{\left(K_{S}C_{e}\right)}^{n_{S}}}$$

The Redlich–Peterson model was expressed as Equation (6):

(6) $$q_{e}=\frac{K_{R}C_{e}}{\left(1+a_{R}{C_{e}}^{\beta }\right)}$$

In these equations, $q_{e}$ is the apparent aqueous‐depletion‐based uptake at the 120‐min endpoint (mg g^−1^), and $C_{e}$ is the corresponding residual aqueous Cr (VI) concentration (mg L^−1^). For the Langmuir model, $q_{\max}$ is the apparent Langmuir capacity parameter (mg g^−1^), and $K_{L}$ is the Langmuir affinity constant (L mg^−1^). For the Freundlich model, $K_{F}$ is the Freundlich adsorption coefficient, with units of (mg g^−1^) (L mg^−1^)^1/n^, and n is the dimensionless Freundlich intensity parameter. For the Sips model, $q_{S}$ is the apparent Sips capacity parameter (mg g^−1^), $K_{S}$ is the Sips affinity constant (L mg^−1^), and $n_{S}$ is the dimensionless Sips heterogeneity exponent. For the Redlich–Peterson model, $K_{R}$ is the Redlich–Peterson coefficient (L g^−1^), $a_{R}$ has units of (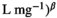, and β is the dimensionless Redlich–Peterson exponent constrained between 0 and 1.

Model parameters were estimated by unweighted nonlinear least squares using the 10 adsorbent‐containing observations in the refined 120‐min dataset. Fits were compared using the coefficient of determination (*R* ^2^), root‐mean‐square error (RMSE), and the small‐sample corrected Akaike information criterion (AICc), for which lower values indicate greater model support after accounting for model complexity. The initial screening dataset was retained as separate supporting evidence of dose–response behavior and observed apparent uptake, but it was not combined with the refined 120‐min dataset for parameter estimation. Supporting fitting data and parameter outputs are provided in Section S5.

### 2.7 Preparation of the Process‐Derived BC/Cr Residue for Asphalt‐Compatible Incorporation

After the adsorption screening experiments, a process‐derived residue was prepared for asphalt‐compatible incorporation. A total of 64 mg of Cr (VI) was added to 40.00 g of BC‐MB, corresponding to a nominal input ratio of 1.6 mg Cr (VI) added per g initial BC. This value describes the mass of Cr (VI) introduced to the preparation; it is not a measured chromium loading, an adsorption efficiency, or a confirmed fraction of an adsorption capacity. Chromium remaining in solution after the 2‐h contact period and total chromium in the recovered solid were not measured.

A total of 40 g of BC‐MB was suspended in 500 mL of distilled water. The suspension was sonicated for 3 min and maintained under magnetic stirring. Then, 160 mL of Cr (VI) stock solution was added, corresponding to the nominal Cr (VI) mass of 64 mg. The mixture was diluted to 750 mL with distilled water, and concentrated sulfuric acid was added to maintain pH below 2. The suspension was stirred for the standardized 120‐min operational contact time used in the adsorption assays.

After contact, the entire suspension was dried at 50°C until the aqueous phase had evaporated. The recovered material was manually disaggregated, macerated, and stored for asphalt binder incorporation. Because dissolved Cr (VI) was not measured after this specific preparation and the recovered solid was not analyzed for total chromium, chromium speciation, mineral phases, elemental composition, or functional groups, the material is referred to hereafter as the process‐derived BC/Cr residue. This term explicitly includes BC, the nominally added chromium, and unresolved material generated or retained during acidification and evaporative drying. Additional mass‐balance, moisture‐content, rehydration, filtration, and control data are provided in Section S6 and Tables S6.1–S6.4.

### 2.8 Asphalt Binder Modification

The process‐derived BC/Cr residue was incorporated into virgin 60–70 penetration‐grade asphalt binder at 0, 3, and 6 wt.% relative to binder mass. The 0 wt.% binder was processed using the same heating and mixing protocol and was used as the processing control. This control was included because high‐temperature mechanical stirring in contact with air may alter the binder independently of residue addition.

For each condition, one 75‐g binder batch was processed. The binder was heated to ensure sufficient fluidity, cooled to equalize thermal history among conditions, and reheated before blending. The process‐derived residue was gradually added to the molten binder. Each binder was mixed at 1250 rpm for 40 min while maintaining an approximate temperature of 150°C–160°C. The processed binders were then cooled before penetration testing, rheological characterization, and asphalt mortar preparation. Detailed residue masses, mixing sequence, and blending conditions are provided in Section S7 and Tables S7.1–S7.2.

### 2.9 Penetration Testing of Asphalt Binders

Penetration testing was conducted to evaluate the consistency of the processed asphalt binders before rheological characterization. The control binder and the binders containing 3 and 6 wt.% process‐derived residue were tested after undergoing the same heating and mixing protocol. Therefore, the penetration results represent the combined effect of processing history and, when applicable, residue incorporation.

Before testing, each binder sample was heated to allow pouring, transferred into penetration containers, cooled, and conditioned at 25°C. Three penetration readings were obtained for each binder condition at separated points on the sample surface, and the average value was used to compare the control and modified binders. Detailed penetration‐test conditions and individual readings are provided in Section S7 and Table S7.3.

### 2.10 Rheological Characterization of Asphalt Binders

The linear viscoelastic response of the processed asphalt binders was evaluated using a dynamic shear rheometer (DSR). Three rheometer specimens were subsampled from each single processed binder batch (control, 3 wt.%, and 6 wt.% residue) and tested to characterize within‐batch measurement consistency. These specimens are not treated as three independent preparation replicates. Frequency sweep tests were conducted to construct master curves of the complex shear modulus magnitude, |G*|.

The applied frequency range was 1–30 Hz, and the temperature sweep included 25°C, 35°C, 45°C, 55°C, 65°C, and 75°C. The reference temperature for master‐curve construction was 25°C. Data obtained at 75°C were not used for asphalt binder master curves because unreliable values were observed at this temperature, likely associated with edge instabilities or torque limitations at low torque thresholds. Therefore, binder master curves were constructed using data collected between 25°C and 65°C.

Frequency–temperature data were horizontally shifted to the reference temperature to obtain reduced‐frequency master curves. The experimentally selected shift factors were compared with values estimated using the Williams–Landel–Ferry (WLF) relationship:

(7) $$\log a_{t}=-\frac{C_{1}\left(T-T_{\mathrm{ref}}\right)}{C_{2}+\left(T-T_{\mathrm{ref}}\right)}$$

where $a_{t}$ is the dimensionless temperature shift factor, T is the test temperature (°C), $T_{\mathrm{ref}}$ is the reference temperature (25°C), $C_{1}$ is the dimensionless WLF coefficient, and $C_{2}$ is the WLF temperature coefficient (°C).

The resulting master curves were used for descriptive comparison of the processed binder conditions over the reduced‐frequency domain. Detailed rheological testing conditions, shift factors, and WLF coefficients are provided in Section S7 and Table S7.4.

### 2.11 Asphalt Mortar Preparation

Asphalt mortars were prepared to evaluate whether binders containing the process‐derived residue could be incorporated into a fine aggregate matrix without strongly altering mortar‐scale rheological response. Two cylindrical mortar specimens were prepared for each binder condition: processed control binder, binder containing 3 wt.% residue, and binder containing 6 wt.% residue.

The mortar gradation was selected to obtain a fine aggregate skeleton suitable for rheological testing in cylindrical specimens. The target asphalt binder content was 9.75% by total mixture mass, with a target air‐void content of 10%. A design bulk specific gravity, G_mb_, of 2.01 was used for mixture proportioning. For each specimen, 20 g of asphalt mortar mixture was prepared, and the target specimen mass was introduced into a metallic cylindrical mold before compression. The complete aggregate gradation, specimen dimensions, and mixture‐proportioning details are provided in Section S8 and Tables S8.1 and S8.2.

Before mixing, aggregate fractions were weighed separately, and the corresponding asphalt binder was heated to mixing temperature. Aggregates and binder were mixed manually until the particles were uniformly coated. The coated mortar was conditioned at elevated temperature, transferred into the heated mold, and compressed using a vertical air‐compression system. After demolding, the specimens were stored in a desiccator, and metallic supports were bonded to both ends using epoxy resin before rheological characterization. Detailed mortar rheological testing conditions, shift factors, WLF coefficients, cyclic recovery data, and replication information are provided in Section S9 and Tables S9.1–S9.7.

### 2.12 Rheological Characterization and Cyclic Recovery Testing of Asphalt Mortars

The rheological behavior of asphalt mortars was evaluated using a DSR equipped with a clamping system for solid cylindrical specimens. Two mortar specimens were tested for each binder condition. Given this small specimen count and the shared binder preparation within each condition, the mortar results are interpreted descriptively rather than as a basis for population‐level inference.

Frequency sweep tests were conducted using the same general temperature–frequency framework applied to the asphalt binders. The applied frequency range was 1–30 Hz, and the test temperatures were 25°C, 35°C, 45°C, 55°C, 65°C, and 75°C. Unlike the binder tests, the 75°C data were retained for the asphalt mortar master curves because the mortar specimens showed stable behavior at this temperature. The reference temperature for master‐curve construction was 25°C. Frequency–temperature data were horizontally shifted to construct reduced‐frequency master curves at the reference temperature, and the experimentally selected shift factors were compared with values estimated using the WLF relationship. Detailed mortar rheological testing conditions, shift factors, WLF coefficients, and replicate‐level master‐curve data are provided in Section S9 and Tables S9.1–S9.5.

In addition to linear viscoelastic characterization, asphalt mortars were subjected to an adapted, nonstandard cyclic creep–recovery test to evaluate deformation recovery after repeated loading. The test was conducted at 40°C using a torsional stress of 10 kPa. Each cycle consisted of 1 s of applied stress followed by 9 s of recovery, repeated for 10 consecutive cycles. The geometry and specimen type differ from the standardized MSCR binder‐film procedure; consequently, the results are reported only as mortar‐cylinder cyclic recovery and should not be compared quantitatively with standard MSCR values from asphalt‐binder literature.

The recovery percentage for each cycle was calculated from the maximum shear strain reached during loading and the residual shear strain measured after the recovery period, according to Equation (8):

(8) $$R_{i}\left(\%\right)=\left(\frac{\gamma_{\max,i}-\gamma_{\mathrm{res},i}}{\gamma_{\max,i}}\right)\times 100$$

where $R_{i}$ is the recovery percentage of cycle i, $\gamma_{\max,i}$ is the maximum shear strain during loading, and $\gamma_{\mathrm{res},i}$ is the residual strain after recovery. For each mortar specimen, recovery was first averaged across the 10 loading–recovery cycles. The two resulting specimen‐level mean recovery values were then averaged to obtain the descriptive mean for each binder condition. The 10 cycles were treated as repeated measurements within each specimen and not as independent replicates. Complete cycle‐by‐cycle recovery data and specimen‐level calculations are provided in Section S9 and Tables S9.2–S9.5.

### 2.13 Data Interpretation and Replication Limits

Rheological results were interpreted descriptively because the experimental structure did not provide sufficient independent replication for reliable inferential testing. Binder frequency points are repeated measurements within rheometer specimens, and the three binder specimens per condition were subsampled from one processed binder batch. Mortar testing included only two specimens per condition. Accordingly, frequency points were not treated as independent experimental units, and no revised ANOVA or post hoc hypothesis tests were used to infer equivalence or differences among formulations.

The revised analysis therefore reports replicate/specimen counts, individual or averaged penetration values, overlapping master‐curve trends, within‐condition variability where available, and cycle‐by‐cycle recovery descriptively. This approach avoids pseudoreplication and is consistent with the proof‐of‐concept objective of determining whether incorporation produced large, readily observable incompatibilities under the tested laboratory conditions.

## 3 Results and Discussion

### 3.1 BC Production Yield and Particle‐Size Reduction

**FIGURE 1:**
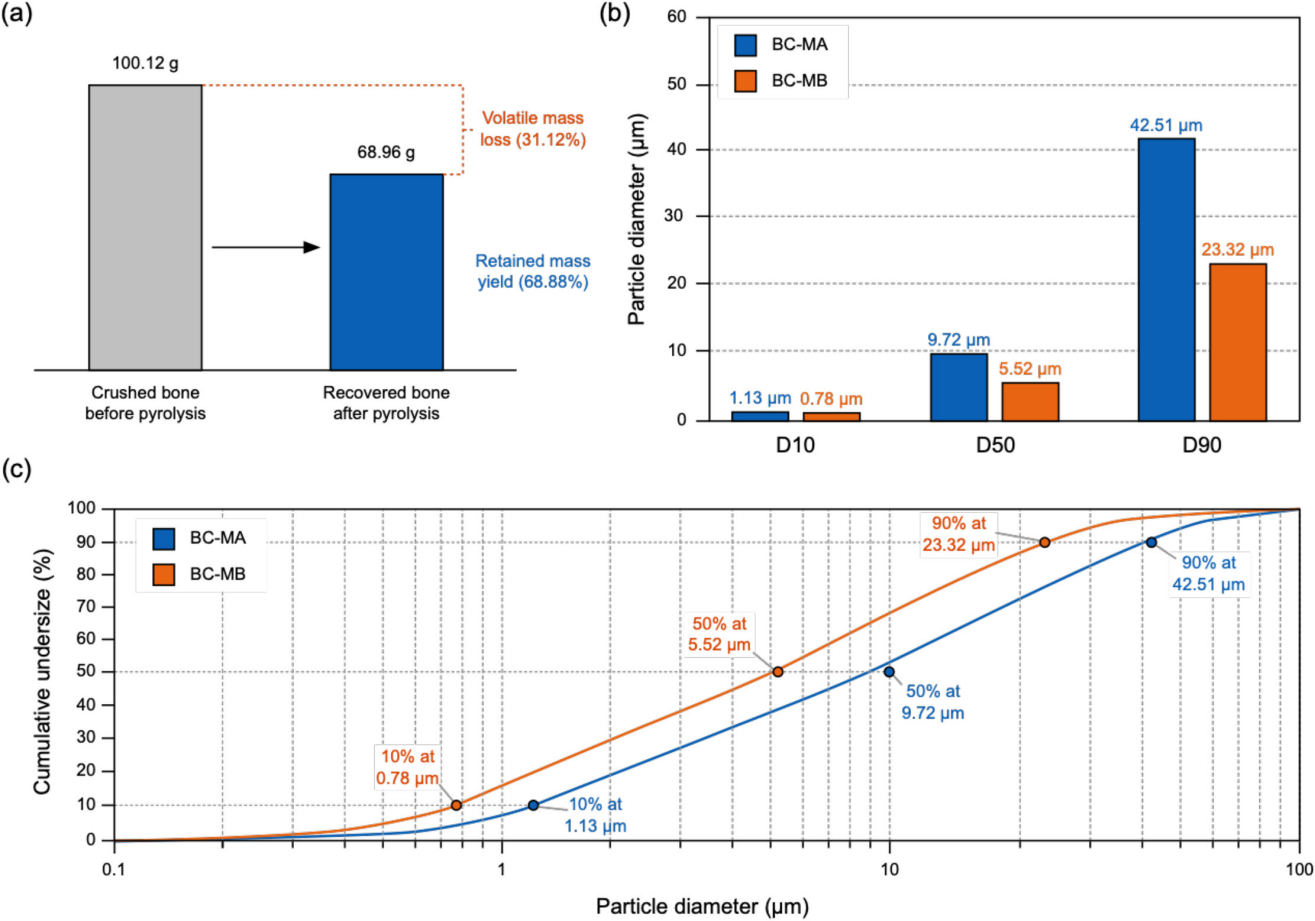
Bone char production yield and particle‐size reduction during sequential milling. (a) Mass balance for thermal conversion of pretreated crushed bovine bone under nitrogen, showing recovered bone char and volatile mass loss. (b) Characteristic particle diameters measured by laser diffraction for BC‐MA and BC‐MB after sonication‐assisted dispersion. (c) Cumulative undersize comparison for D10, D50, and D90. BC‐MA corresponds to bone char after high‐energy roller milling, whereas BC‐Mb corresponds to bone char after sequential roller milling and planetary ball milling.

Thermal conversion under nitrogen produced a recoverable mineral–carbonaceous material from bovine bone waste. From 100.12 g of crushed bone introduced into the furnace, 68.96 g of solid material was recovered after pyrolysis, corresponding to a retained mass yield of 68.9% and an estimated volatile mass loss of 31.1% (Figure 1a). This retained fraction is consistent with the mineral‐rich nature of bone‐derived chars, in which calcium‐phosphate phases remain after removal of moisture, organic matter, and volatile components (Hart et al. 2023; Medellín Castillo et al. 2023; Patel et al. 2015). The recovered material was subsequently processed by sequential mechanical milling to obtain a finer BC fraction suitable for adsorption testing and asphalt‐compatible incorporation.

Sequential milling shifted the particle‐size distribution toward smaller diameters (Figure 1b,c). The material obtained after high‐energy roller milling, referred to as bc‐MA, showed D10, D50, and D90 values of 1.13, 9.72, and 42.51 μm, respectively. After additional planetary ball milling, the BC‐MB fraction showed lower D10, D50, and D90 values of 0.78, 5.52, and 23.32 μm, respectively. The reduction in median particle diameter from 9.72 to 5.52 μm, together with the decrease in D90 from 42.51 to 23.32 μm, confirms that the second milling stage reduced the coarser fraction and improved particle‐size conditioning. Therefore, BC‐MB was selected as the working material for Cr (VI) adsorption and asphalt‐compatible incorporation experiments.

Although BC‐MB included a submicron D10 fraction, its median particle diameter remained in the micrometer range. The material is therefore described throughout this study as fine‐milled BC with a submicron fraction, rather than as an ultrafine or nanoscale adsorbent. Laser diffraction data alone do not demonstrate uniformly nanometric behavior or a specific adsorption mechanism. Detailed particle‐size metrics are provided in Tables S2.1 and S2.2.

### 3.2 Contact‐Time Screening and Operational Contact Time

**FIGURE 2:**
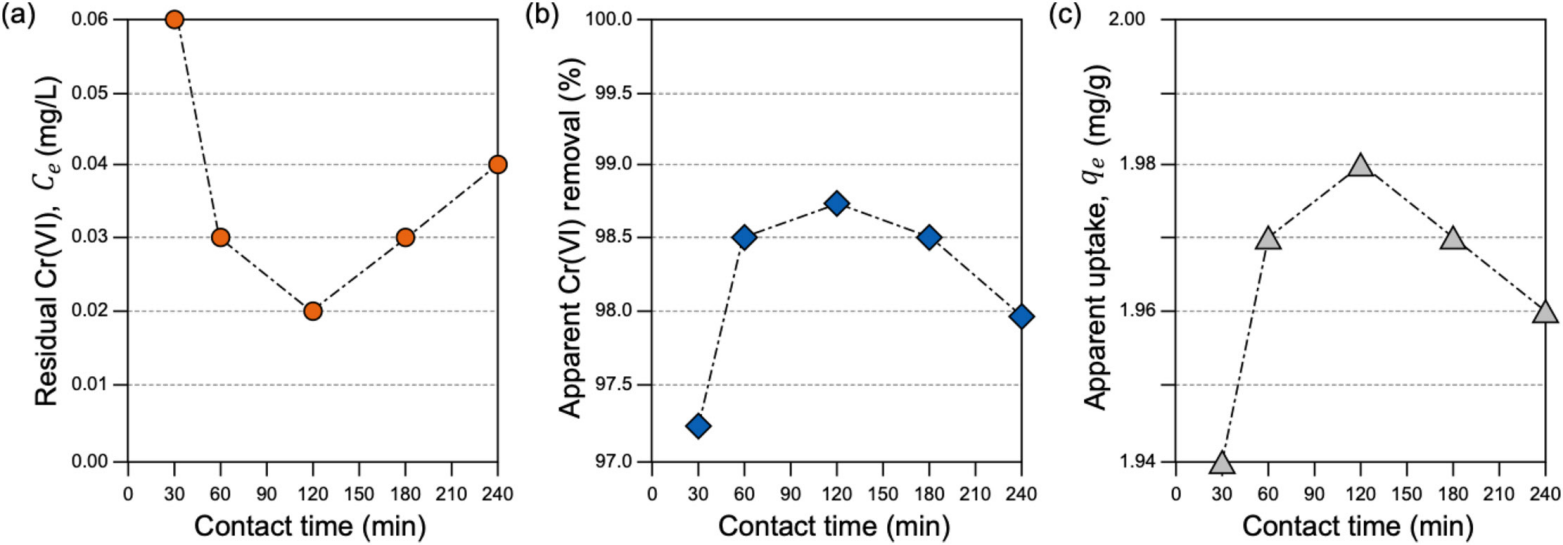
Contact‐time screening for apparent aqueous Cr (VI) removal by fine‐milled bone char. (a) Archived residual Cr (VI) concentration versus contact time for samples containing 2 mg L^−1^ initial Cr (VI) and 1 g L^−1^ BC. (b) Calculated apparent Cr (VI) removal efficiency. (c) Apparent aqueous‐depletion‐based uptake, $q_{e}$. The archived residual concentrations were consistently low from 60 to 240 min; however, the sample‐equivalent lower calibration bound for this assay could not be reconstructed from the available dilution records, and only one BC‐containing observation was available per contact time. Therefore, 120 min was retained only as a standardized operational contact time and not as a demonstrated equilibrium time.

The contact‐time screening assay showed rapid apparent aqueous Cr (VI) removal under the tested acidic conditions. At $C_{0}$ = 2 mg L^−1^ and a BC dose of 1 g L^−1^, the archived measurement after 30 min was 0.06 mg L^−1^, corresponding to a calculated apparent removal of 97.2% (Figure 2a,b). Archived residual concentrations from 60 to 240 min ranged from 0.02 to 0.04 mg L^−1^, corresponding to calculated apparent removals of 98.0%–98.8%. However, the available experimental records do not document the aliquot volume used for dilution in this contact‐time assay, so the sample‐equivalent lower calibration bound cannot be reconstructed reliably. In addition, the assay included only one BC‐containing observation at each contact time. Therefore, the small differences among the very low residual concentrations measured from 60 to 240 min cannot be interpreted confidently as kinetic differences. A 120‐min contact period was retained as a standardized operational time for subsequent experiments because it lays within this region of consistently low archived residual Cr (VI) measurements, not because the experiment statistically demonstrated equilibrium.

The process blanks varied over time, with archived Cr (VI) concentrations of 1.86–2.09 mg L^−1^ relative to the nominal 2 mg L^−1^ starting concentration, corresponding to apparent changes from +7.2% removal to −4.4% removal. This variation indicates measurable uncertainty associated with solution preparation, dilution, color development, and instrumental response. Using each time‐matched blank as the reference, the calculated apparent removal for the corresponding BC‐containing sample remained between 97.0% and 98.9%. Thus, the magnitude of the aqueous Cr (VI) decrease in the BC‐containing samples was substantially greater than the variability observed in the blanks. However, the blank variability, the absence of independent replicates, and the unresolved sample‐equivalent lower calibration bound reinforce that small differences among the later contact times should not be interpreted quantitatively. The time‐matched blank sensitivity calculation is provided in Table S4.7.

The contact‐time experiment was not designed for mechanistic kinetic modeling. It contained only one BC‐containing observation per contact time, no measurements before 30 min, and very low archived residual Cr (VI) concentrations for which the sample‐equivalent lower calibration bound cannot be reconstructed from the available dilution records. Fitting PFO, PSO, Elovich, or intraparticle‐diffusion models to these five points would therefore provide poorly identifiable parameters and a misleading impression of mechanistic resolution. The results are used only to select a standardized operational contact time. Denser early‐time sampling, independent replication, complete analytical dilution records, pH tracking, chromium speciation, and evaluation of competing ions or real tannery wastewater would be required for defensible kinetic‐model discrimination (Hart et al. 2023; Qasem et al. 2021; Rajapaksha et al. 2022; Ranjbar et al. 2018).

### 3.3 Dose–Response Behavior

Using the standardized 120‐min operational contact time, dose–response adsorption assays evaluated the effects of BC dose and initial Cr (VI) concentration on residual concentration, apparent removal efficiency, and apparent uptake. The initial screening assay indicated near‐complete aqueous Cr (VI) removal under favorable low‐concentration conditions: At $C_{0}$ = 2 mg L^−1^, BC doses of 1–10 g L^−1^ produced calculated apparent removals of 98.1%–99.7%. However, these samples were analyzed using a fivefold dilution, for which the lowest nonzero calibration standard corresponds to 0.10 mg L^−1^ in the original sample. Therefore, screening measurements below this sample‐equivalent concentration are interpreted only as indicating very low residual Cr (VI), rather than as quantitatively distinguishable concentrations or removal percentages. The refined dose–response assay provided substantially better analytical resolution and was therefore used for model fitting and quantitative interpretation.

**FIGURE 3:**
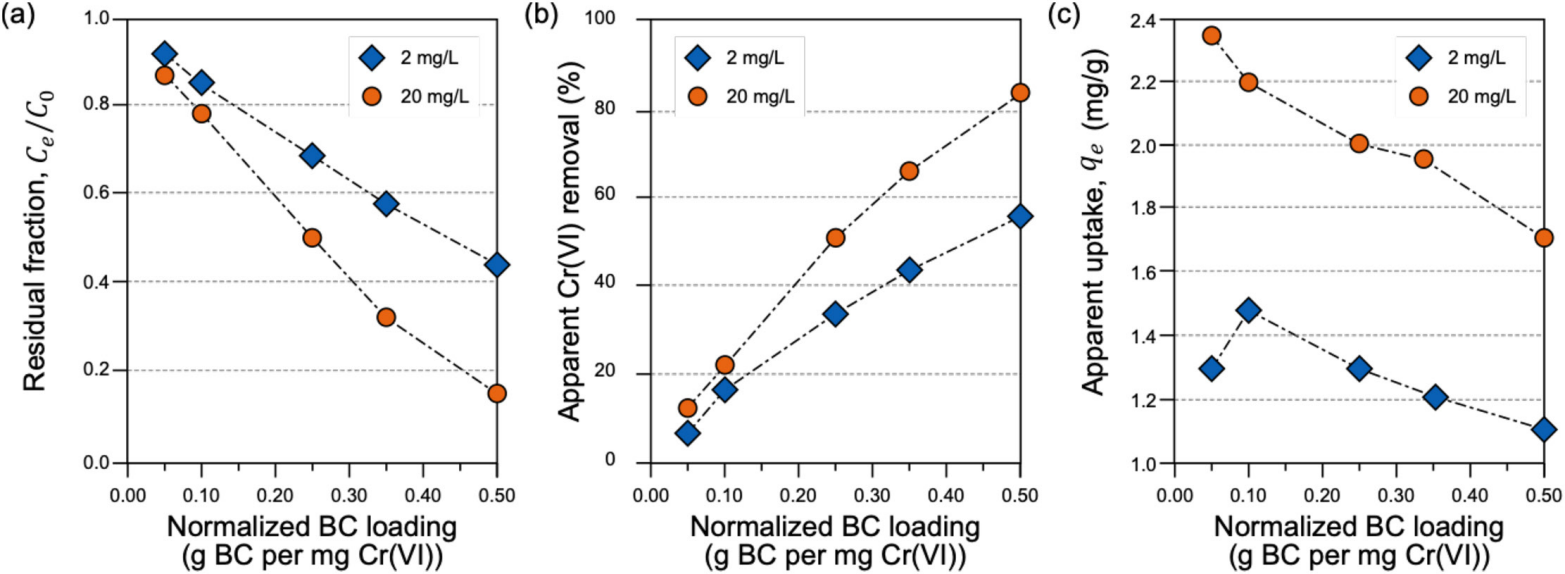
Dose–response behavior for apparent aqueous Cr (VI) removal by fine‐milled bone char in the refined 120‐min assay. (a) Residual Cr (VI) fraction, $C_{e}/C_{0}$, as a function of normalized BC loading. (b) Apparent Cr (VI) removal efficiency. (c) Apparent aqueous‐depletion‐based uptake, qₑ. Normalized BC loading was calculated as BC dose divided by initial Cr (VI) concentration. All samples were contacted for the standardized 120‐min operational contact time.

In the refined assay, apparent Cr (VI) removal increased with BC dose for both initial concentrations tested. For $C_{0}$ = 2 mg L^−1^, residual Cr (VI) decreased from 1.87 to 0.88 mg L^−1^ as the BC dose increased from 0.1 to 1.0 g L^−1^, corresponding to 6.4%–55.9% removal (Figure 3a,b). For $C_{0}$ = 20 mg L^−1^, residual Cr (VI) decreased from 17.65 to 3.04 mg L^−1^ as BC dose increased from 1 to 10 g L^−1^, corresponding to 11.7%–84.8% removal. After accounting for the documented dilution factors, all residual concentrations in the refined dataset fell within the nonzero calibration range used for UV–Vis quantification. The response therefore depended on both BC dose and the initial contaminant‐to‐adsorbent ratio.

Apparent uptake, qₑ, must be interpreted separately from removal efficiency. In the refined dataset, qₑ ranged from 1.12 to 1.48 mg g^−1^ for the 2 mg L^−1^ series and from 1.70 to 2.35 mg g^−1^ for the 20 mg L^−1^ series (Figure 3c). In the separate screening assay, the highest observed $q_{e}$ was 4.39 mg g^−1^ at $C_{0}$ = 20 mg L^−1^ and 1 g L^−1^ BC, despite only 21.9% removal. Thus, 2 mg g^−1^ should not be interpreted as a maximum adsorption capacity. Removal percentage and qₑ answer different questions: Increasing adsorbent dose can improve residual water quality while decreasing the contaminant mass removed per gram of adsorbent.

The contrast between the screening and refined assays shows why maximum removal efficiency alone is insufficient for evaluating this material. Apparent removal is not an intrinsic fixed property of BC; it depends on initial concentration, dose, residual concentration, pH, contact conditions, and solution chemistry. Likewise, a single observed apparent $q_{e}$ value is not equivalent to a maximum adsorption capacity or a validated equilibrium parameter. Reported Cr (VI) capacities in recent waste‐derived adsorbent literature vary widely with precursor, chemical modification, pH, concentration range, model choice, and analytical design (Hart et al. 2023; Loc and Phuong 2025; Nguyen et al. 2024; Qasem et al. 2021; Rajapaksha et al. 2022; Ranjbar et al. 2018; Yu and Yang 2024; Zou et al. 2023). The present fine‐milled, unfunctionalized BC therefore should be positioned as a low‐complexity proof‐of‐concept material rather than as a high‐capacity competitor to engineered Cr (VI) adsorbents.

For treatment‐oriented interpretation, the selected BC dose must balance residual Cr (VI), adsorbent consumption, and the quantity and composition of the residual solid requiring management. This trade‐off is particularly important here because the material recovered after Cr (VI)/acid exposure and drying was subsequently tested in asphalt‐compatible matrices. Complete screening and refined dose–response data are provided in Tables S4.5 and S4.6.

### 3.4 Adsorption Model Fitting

**FIGURE 4:**
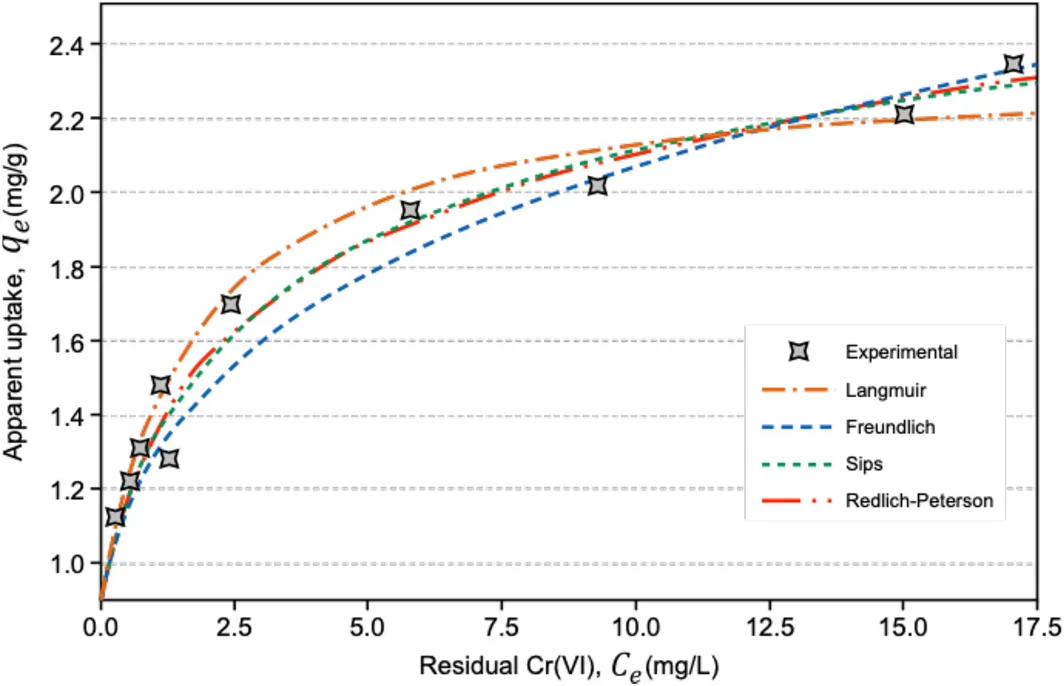
Nonlinear isotherm model fitting for the refined 120‐min Cr (VI) dose–response dataset. Experimental apparent uptake values, $q_{e}$, are plotted against residual Cr (VI), $C_{e}$, together with nonlinear Langmuir, Freundlich, Sips, and Redlich–Peterson curves. Sips and Redlich–Peterson produced slightly lower residual errors, whereas Freundlich had the lowest AICc and therefore the strongest parsimonious support. Models are interpreted as empirical descriptors rather than as evidence of equilibrium or a specific removal mechanism.

The refined 120‐min dose–response dataset was fitted using nonlinear Langmuir, Freundlich, Sips, and Redlich–Peterson isotherm models. Direct nonlinear fitting avoids distortions introduced by linear transformations and allows the models to be compared on the original $q_{e}$ scale (Tansomros et al. 2025). Because equilibrium was not independently demonstrated, the fitted models are interpreted as empirical descriptions of the observed $q_{e}-C_{e}$ relationship at the standardized 120‐min contact time rather than as validated equilibrium relationships. The experimental data, fitted curves, and comparison metrics are summarized in Table 1 and Figure 4; supporting parameter outputs are provided in Section S5.

**TABLE 1:** Nonlinear isotherm‐model parameters obtained from the refined 120‐min Cr (VI) dose–response dataset.

Model	Key fitted parameters	*R* ^2^	RMSE (mg g^−1^)	AICc
Langmuir	$q_{\max}$ = 2.35 mg g^−1^; $K_{L}$ = 0.884 L mg^−1^	0.955	0.089	−42.59
Freundlich	$K_{F}$ = 1.223 (mg g^−1^) (L mg^−1^)^1/n^; *n* = 4.423	0.964	0.079	−44.93
Sips	$q_{S}$ = 3.09 mg g^−1^; $K_{S}$ = 0.403 L mg^−1^; $n_{S}$ = 0.536	0.974	0.068	−43.69
Redlich–Peterson	$K_{R}$ = 4.32 L g^−1^; $a_{R}$ = 2.70 (L mg^−1^)^*β*^; β = 0.864	0.975	0.067	−43.96

*Note:* Model parameters were obtained from the refined 120‐min dose–response dataset. Because equilibrium was not independently demonstrated, the fitted parameters are interpreted as empirical descriptors of the observed $q_{e}-C_{e}$ relationship at the standardized contact time. In particular, $q_{\max}$ and $q_{S}$ are fitted capacity parameters and should not be interpreted as experimentally demonstrated maximum adsorption capacities.

All four models described the small refined dataset reasonably well, but the ranking depended on whether residual error or parsimony was emphasized. Redlich–Peterson and Sips gave the highest *R* ^2^ values (0.975 and 0.974) and the lowest RMSE values (0.067 and 0.068 mg g^−1^), respectively. Freundlich gave a slightly lower *R* ^2^ of 0.964 and RMSE of 0.079 mg g^−1^ but had the lowest AICc (−44.93) because it used one fewer fitted parameter than Sips or Redlich–Peterson. Langmuir gave *R* ^2^ = 0.955, RMSE = 0.089 mg g^−1^, and an apparent $q_{\max}$ parameter of 2.35 mg g^−1^. Because the separate screening dataset contained an observed apparent $q_{e}$ of 4.39 mg g^−1^, the fitted Langmuir $q_{\max}$ parameter from the refined 120‐min dataset should not be interpreted as an experimentally demonstrated maximum adsorption capacity.

The nonlinear model comparison does not resolve an adsorption mechanism. Freundlich receives the greatest AICc support because it provides a parsimonious description of the available data, whereas the three‐parameter Sips and Redlich–Peterson models reduce residual error slightly at the cost of greater parameter uncertainty. Bone‐derived chars can contain calcium‐phosphate mineral domains, carbonaceous regions, pores, and chemically distinct surface sites (Hart et al. 2023; Medellín Castillo et al. 2023; C. Rojas‐Mayorga et al. 2016), but the present study did not measure BET surface area, crystallographic phases, ${\mathrm{pH}}_{\mathrm{pzc}}$, zeta potential, functional groups, or chromium speciation after contact. Accordingly, model fit is treated as empirical description rather than evidence for monolayer adsorption, chemisorption, surface heterogeneity, reduction, or any other specific mechanism (Nguyen et al. 2024; Yu and Yang 2024).

The apparent uptake values obtained here are modest relative to many engineered or chemically modified Cr (VI) adsorbents. That comparison is expected because the present BC was not chemically activated or functionalized with metal oxides, polymers, or magnetic phases. Recent waste‐derived adsorbents report much larger fitted Cr (VI) capacities under optimized conditions, but those values are not directly transferable because precursor composition, pH, concentration range, surface modification, competing ions, model selection, and analytical methods differ among studies (Loc and Phuong 2025; Nguyen et al. 2024; Ranjbar et al. 2018; Yu and Yang 2024; Zou et al. 2023). Table 2 therefore provides context rather than a performance ranking. The contribution of the present material is the lower complexity capture‐to‐residue‐management proof‐of‐concept, not record adsorption capacity.

**TABLE 2:** Selected Cr (VI) adsorption capacities and apparent uptake values reported for waste‐derived adsorbents.

Study	Adsorbent	Modification/test context	Reported Cr (VI) capacity or apparent uptake	Interpretive use here
Present study	Bovine bone char	Fine‐milled; no chemical activation; acidic synthetic water	Observed apparent *q_e_* up to 4.39 mg g^−1^; apparent Langmuir *q_max_* parameter = 2.35 mg g^−1^ for the refined 120‐min dataset	Low‐complexity proof‐of‐concept; values are dataset‐dependent
Ranjbar et al. (2018)	Bone char–ZnO composite	ZnO‐functionalized bone char; optimized Cr (VI) adsorption	78.59 mg g^−1^	Direct bone char Cr (VI) comparison but chemically functionalized
Zou et al. (2023)	Carbonized rice husk + hydroxyapatite	Hydroxyapatite‐modified waste biochar	14.28 mg g^−1^	Recent waste‐derived Cr (VI) adsorbent
Nguyen et al. (2024)	Corncob biochar	Unmodified corncob‐derived biochar; pH 2	38.13 mg g^−1^	Recent unmodified waste‐derived Cr (VI) biochar
Yu and Yang (2024)	Sludge biochar + Fe–Mn oxides	Metal‐oxide‐loaded sludge biochar	172.3 mg g^−1^	Shows capacity gains achievable with engineered surface chemistry
Loc and Phuong (2025)	Mimosa biochar + hydroxyapatite	Hydroxyapatite‐modified biochar	67.68 mg g^−1^	Recent HAp‐containing Cr (VI) adsorbent; not bone char

*Note:* Reported capacities are not directly comparable across studies because precursor chemistry, activation, pH, initial concentration, dose, analytical method, and fitted model differ. The table provides context rather than a performance ranking.

Overall, the isotherm models provide empirical descriptions of the tested 120‐min synthetic‐water dataset but should not be used as equilibrium or design parameters for real tannery wastewater. Broader concentration ranges, independent replication, pH and ionic strength variation, competing‐ion tests, real wastewater, chromium speciation, and direct surface characterization are required before mechanistic or treatment‐design claims can be supported.

### 3.5 Composition‐Related Implications of the Process‐Derived Residue

**FIGURE 5:**
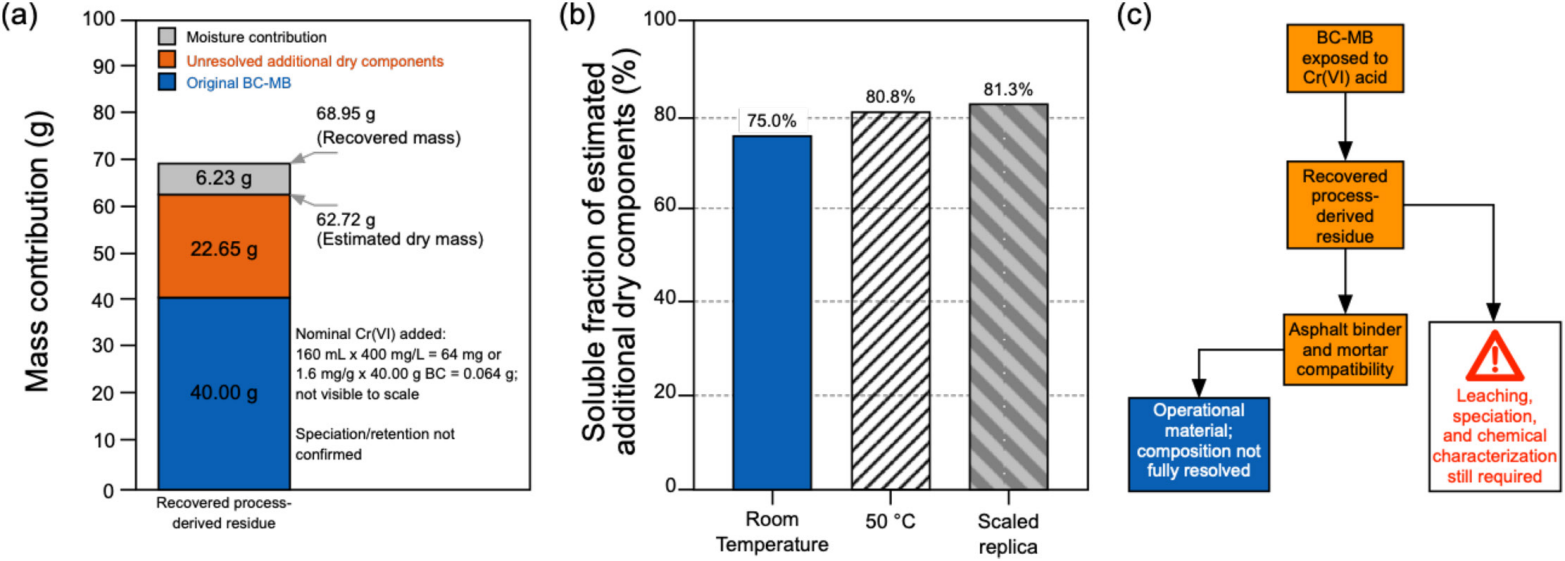
Composition‐related interpretation of the process‐derived BC/Cr residue. (a) Reconstructed mass distribution after preparation, showing the initial 40.00 g BC‐MB, 22.65 g unresolved additional dry material, and estimated moisture contribution. The nominal 64 mg Cr (VI) input is not visible to scale and was not measured as retained chromium. (b) Water‐soluble fraction of the estimated additional dry material during rehydration at room temperature, 50°C, and in the scaled replica. (c) Interpretation boundary: The complete process‐derived residue was used in asphalt compatibility tests, and leaching, chromium speciation, and chemical/mineralogical characterization remain required.

Preparation of the process‐derived BC/Cr residue produced a marked mass increase after evaporative drying. The preparation contained 40.0043 g of BC‐MB and a nominal 64 mg of added Cr (VI), corresponding to 1.6 mg Cr (VI) added per g initial BC. This ratio is an input calculation only; retained chromium was not measured. After acidic contact and drying, 68.95 g of material was recovered. Moisture analysis indicated 9.0% water, corresponding to an estimated dry mass of 62.72 g. After subtracting the original Bc mass and the nominal Cr input, approximately 22.65 g of dry material remained unexplained by those two inputs. This unresolved mass is a major component of the material actually introduced into the asphalt experiments (Figure 5a).

The recovered material therefore cannot be described as BC carrying only adsorbed chromium. Potassium dichromate and sulfuric acid were present during preparation, and the BC mineral phase was exposed to pH < 2 for 120 min before the complete aqueous phase was evaporated. Plausible contributors to the additional dry mass include acid‐ or potassium‐containing residues and dissolution/reprecipitation products from the mineral fraction of BC, but the present dataset cannot identify or quantify these species. The acid/dichromate control without BC retained approximately 1.1 mL of residual liquid, with a recorded mass of 1.9967 g, after sequential drying at 50°C and 103°C, whereas the BC‐containing scaled replica produced 4.71 g of dried material. This contrast indicates that the mass gap cannot be assigned solely from the no‐BC control. XRD, FTIR, elemental analysis/ICP, SEM–EDS, or XPS would be required to resolve the residue composition and determine where chromium is located.

Rehydration and filtration tests further showed that much of the unresolved additional dry material was water‐soluble. At room temperature, the soluble mass represented 75.0% of the estimated additional components; at 50°C, it represented 80.8%, and the scaled replica gave 81.3%. These results do not identify the dissolved species, but they demonstrate that the process‐derived residue contains a substantial water‐soluble fraction. Detailed mass‐balance, moisture, rehydration, filtration, and control observations are provided in Tables S6.1–S6.4.

This finding materially limits environmental interpretation of the asphalt experiments. If a similar residue were incorporated into pavement and later contacted by runoff, wet–dry cycles, cracking, abrasion, or end‐of‐life recycling, soluble chromium‐bearing, potassium‐containing, sulfate‐containing, or other acid‐derived species could potentially be mobilized (Raj et al. 2022; Wang and Wang 2022; Zein et al. 2022). The asphalt phase is therefore evaluated only as a preliminary processing‐compatibility matrix. The study does not demonstrate chromium immobilization, safe disposal, or environmental reuse. Direct leaching, chromium speciation, total‐chromium analysis, and residue mineralogical/chemical characterization are prerequisite follow‐up tests.

Overall, the composition‐related results define the boundary condition for the asphalt portion of the study: The tested material is the complete process‐derived residue produced by acidic Cr (VI) exposure and evaporative drying, not pristine BC and not a chemically verified Cr‐loaded adsorbent. Any observed asphalt response therefore belongs to that unresolved residue as a whole.

### 3.6 Asphalt Binder Compatibility

**FIGURE 6:**
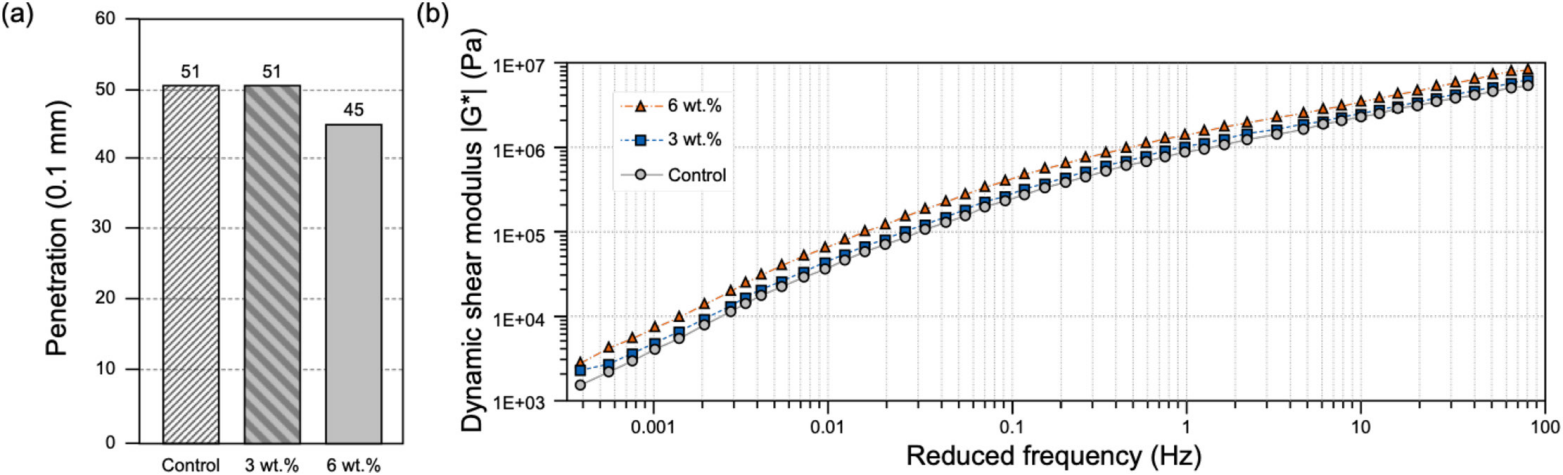
Descriptive asphalt binder response after incorporation of the process‐derived BC/Cr residue. (a) Penetration values for the processed control and 3 and 6 wt.% residue conditions; bars show the mean of three within‐sample technical readings. (b) Average dynamic shear modulus master curves at 25°C for the three processed binder conditions. No inferential *p*‐values are reported because the binder specimens were subsamples from one processed batch per condition.

Penetration testing showed the same mean value for the processed control and 3 wt.% residue conditions, 51 (0.1 mm), whereas the 6 wt.% condition averaged 45 (0.1 mm) (Figure 6a). The 6 wt.% value is about 12% lower than the processed control, indicating a descriptive stiffening trend at the higher residue dosage. Because the three penetration readings were taken at separated points on one processed binder sample per condition, they characterize within‐sample consistency rather than independent treatment replication. Individual readings are provided in Table S7.3.

DSR master curves at a 25°C reference temperature also showed broad overlap among the processed control, 3 wt.%, and 6 wt.% binder conditions (Figure 6b). Minor separation occurred across portions of the reduced‐frequency domain, but there was no large systematic displacement of the modified curves relative to the control. Because each condition was represented by one processed binder batch and three rheometer subsamples from that batch, this visual overlap is interpreted as a screening‐level compatibility observation, not as statistical evidence of equivalence. Binder WLF coefficients and shift factors are provided in Table S7.4.

The revised analysis does not report ANOVA *p*‐values for binder master curves. Frequency points within a master curve are repeated measurements from the same specimen, and the three binder specimens per condition were derived from a single processed batch; treating those points as independent observations would constitute pseudoreplication. The previous pairwise ANOVA screening results have therefore been removed from both the manuscript and Supporting Information.

From a materials‐processing perspective, the absence of a large descriptive shift in the binder master curves indicates that the process‐derived residue could be blended at 3 and 6 wt.% without an obvious loss of rheological compatibility under the tested conditions. This result should not be interpreted as proof of material equivalence, binder replacement, long‐term performance, or environmental safety. Storage stability, aging, workability, mixture‐level performance, and leaching remain necessary before practical use can be considered (Ashish et al. 2016; Huang et al. 2024; Xu et al. 2019).

### 3.7 Asphalt Mortar Response and Cyclic Recovery Behavior

**FIGURE 7:**
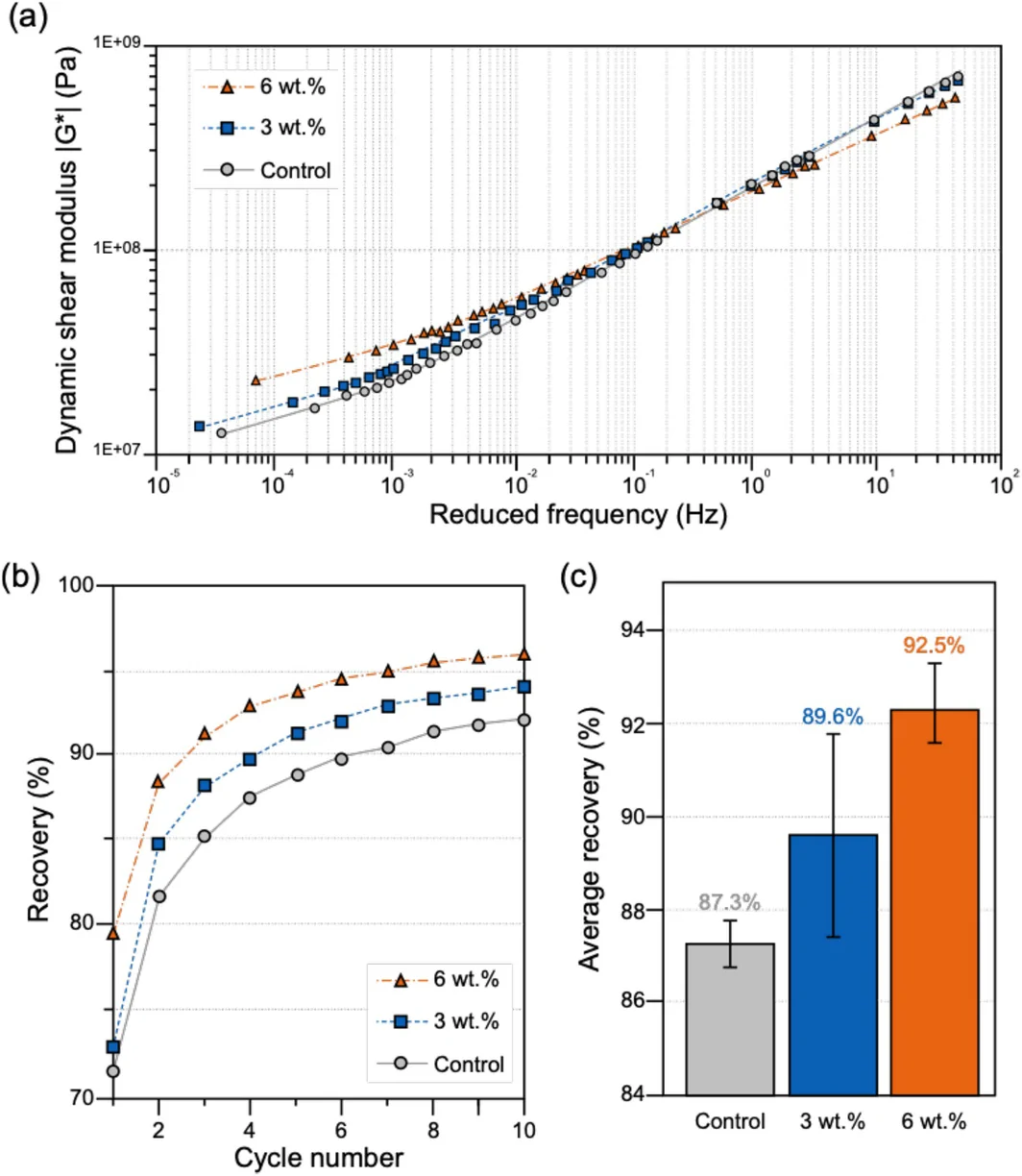
Descriptive asphalt mortar response after incorporation of the process‐derived BC/Cr residue. (a) Average dynamic shear modulus master curves at 25°C for control, 3 and 6 wt.% residue conditions. (b) Cycle‐by‐cycle recovery during the adapted non‐standard mortar‐cylinder creep–recovery test at 40°C and 10 kPa; lines show the mean of two specimens per condition. (c) Overall average recovery across 10 cycles; bars show the mean of the two mortar specimens per condition and error bars represent the between‐specimen standard deviation (*n* = 2). These recovery values are not standard binder MSCR results.

The asphalt mortar master curves were broadly comparable across the processed control, 3 and 6 wt.% residue conditions (Figure 7a). Some separation was visually apparent, particularly for the 6 wt.% condition over portions of the reduced‐frequency range, but only two mortar specimens were available per condition. The revised analysis therefore does not report ANOVA *p*‐values or infer statistical equivalence/difference. The master curves are interpreted as descriptive evidence that residue incorporation did not produce a large rheological incompatibility within this small proof‐of‐concept specimen set.

The nonstandard mortar‐cylinder cyclic creep–recovery test showed a clearer dose‐related trend than the master‐curve comparison. Recovery increased across the first cycles and approached a more stable range for all conditions (Figure 7b). For each mortar specimen, recovery was first averaged across the 10 loading–recovery cycles. The two resulting specimen‐level values were then averaged within each condition, giving mean recoveries of 87.3% for the control, 89.6% for 3 wt.% residue, and 92.5% for 6 wt.% residue (Figure 7c). These descriptive values indicate higher recovery in the tested residue‐containing specimens, but the small specimen count and nonstandard test geometry preclude population‐level inference or direct comparison with standardized binder MSCR literature. Mixture design, WLF coefficients, test conditions, and cycle‐by‐cycle values are provided in Sections S8 and S9.

The recovery trend may reflect filler‐like or binder–particle effects of the process‐derived residue, but the present experiments cannot assign a mechanism (Ashish et al. 2016; Bhat and Mir 2021; Huang et al. 2024). Mortar microstructure, particle dispersion, binder–particle adhesion, final air‐void content, and specimen‐specific volumetrics were not measured. Moreover, the tested residue contains unresolved soluble material in addition to BC. The cyclic recovery result should therefore be interpreted as a screening signal that motivates better‐replicated, standardized mechanical testing rather than as evidence of improved rutting, fatigue, or pavement durability.

Variability between the two mortar specimens further indicates sensitivity to preparation and volumetric control. Theoretical mixture proportions were specified, but final specimen air voids, bulk specific gravity, aggregate coating, and internal structure were not experimentally verified. These factors should be controlled in future work before attributing recovery differences to residue dosage.

Overall, the mortar experiments support only a preliminary compatibility conclusion: The process‐derived residue could be incorporated into the tested fine aggregate matrix without a large descriptive disruption of the master curves, while average cyclic recovery was higher at increasing residue dosage in this small specimen set. The study does not demonstrate full‐asphalt mixture performance, long‐term durability, rutting or fatigue resistance, moisture resistance, aging stability, or chromium containment.

### 3.8 Integrated Implications and Boundary Conditions

The combined results support an experimentally bounded sequence linking fine‐milled BC contact with Cr (VI)‐containing synthetic water, recovery of an unresolved process residue, and asphalt‐compatible processing of that residue. Under the tested conditions, BC produced large apparent aqueous Cr (VI) decreases, dose‐dependent responses were observed in the refined 120‐min assay, and nonlinear isotherm models provided empirical descriptions of the limited dataset. The resulting process‐derived residue could then be incorporated into asphalt binder and mortar without large descriptive shifts in the measured master curves, while the nonstandard mortar‐cylinder recovery metric increased with residue dosage. These observations establish proof‐of‐concept processing compatibility, not treatment validation or contaminant containment.

The adsorption component has several important boundaries. The experiments used synthetic Cr (VI) solutions under controlled acidic conditions, so the observed performance should not be extrapolated directly to tannery wastewater, where pH, ionic strength, organic matter, suspended solids, Cr (III), competing anions, sulfides, chlorides, oils, dyes, and other process chemicals may alter behavior (Qasem et al. 2021; Rajapaksha et al. 2022). Only dissolved Cr (VI) was quantified; total chromium and Cr (III) were not measured. Consequently, aqueous Cr (VI) depletion cannot establish chromium speciation, distinguish adsorption from possible redox transformation, or prove where chromium resides in the recovered solid (Nguyen et al. 2024; Yu and Yang 2024). Surface chemistry, ${\mathrm{pH}}_{\mathrm{pzc}}$, zeta potential, functional groups, and mineral phases were also not measured, so mechanistic assignments are intentionally avoided.

A second boundary concerns the identity and environmental behavior of the process‐derived residue. Evaporative drying retained not only BC and nominally introduced chromium but also 22.65 g of unresolved additional dry material, of which approximately 75%–81% was water‐soluble in rehydration tests. The asphalt experiments therefore did not evaluate a chemically defined Cr‐loaded BC adsorbent. They evaluated the complete residue generated by the preparation route. Direct leaching, total chromium, Cr (VI)/Cr (III) speciation, XRD/FTIR/SEM–EDS or equivalent compositional analysis, wet–dry cycling, aging, abrasion, and end‐of‐life exposure are required before any immobilization, reuse, or safe disposal claim can be considered (Raj et al. 2022; Wang and Wang 2022; Zein et al. 2022).

The asphalt results are likewise binder‐ and mortar‐scale screening observations rather than pavement‐performance validation. The binder and mortar master curves did not show large descriptive incompatibilities, but the experimental design provided only one processed binder batch per condition and two mortar specimens per condition. Field performance depends on storage stability, mixing and compaction, aggregate coating, air‐void structure, rutting, fatigue, moisture susceptibility, oxidative aging, and environmental exposure (Ashish et al. 2016; Huang et al. 2024; Xu et al. 2019). Better‐replicated full‐mixture testing is therefore required before performance claims.

The main novelty of the study is thus the transparent linkage of three stages that are often studied separately: apparent aqueous Cr (VI) removal by a waste‐derived adsorbent, mass‐balance characterization of the recovered process residue, and preliminary asphalt‐compatible processing. The work identifies both a possible materials‐management pathway and the critical evidence still missing for that pathway to be environmentally credible (Raj et al. 2022; Zein et al. 2022). The minimum additional validation needs are summarized in Table S10.1.

## 4 Conclusions

This study evaluated a laboratory proof‐of‐concept linking apparent aqueous Cr (VI) removal by fine‐milled bovine BC with preliminary asphalt‐compatible processing of the residue recovered after acidic Cr (VI) exposure and evaporative drying. The work does not demonstrate final treatment, chromium immobilization, or safe disposal.

BC‐MB had D10 = 0.78 μm, D50 = 5.52 μm, and D90 = 23.32 μm and is therefore best described as fine‐milled BC with a submicron fraction rather than as an ultrafine or nanoscale adsorbent. In the contact‐time screening assay, the archived measurements indicated > 97% calculated apparent Cr (VI) removal after 30 min and consistently very low residual concentrations thereafter. However, the sample‐equivalent lower calibration bound could not be reconstructed from the available dilution records for this assay, and only one BC‐containing observation was available per contact time. Therefore, 120 min was retained only as a standardized operational contact time and not as a statistically demonstrated equilibrium time.

Removal efficiency and apparent uptake depended strongly on initial Cr (VI) concentration and BC dose. The highest observed qₑ was 4.39 mg g^−1^ in the screening dataset; therefore, 2 mg g^−1^ is not a defensible maximum‐capacity value. Nonlinear Langmuir, Freundlich, Sips, and Redlich–Peterson isotherm models all described the refined 120‐min dose–response dataset, with Freundlich receiving the lowest AICc while the three‐parameter models produced slightly lower residual errors. Because equilibrium was not independently demonstrated, these fits are interpreted only as empirical descriptions of the observed qₑ–Cₑ relationship at the standardized contact time and do not establish equilibrium, adsorption capacity, or a specific removal mechanism.

The residue used for asphalt experiments was compositionally unresolved. Starting from 40.0043 g BC and a nominal 64 mg Cr (VI) input, evaporative drying produced an estimated 62.72 g dry residue, leaving approximately 22.65 g of additional dry material beyond the initial BC and nominal Cr input. Rehydration showed that about 75%–81% of this additional material was water‐soluble. The study therefore does not establish chromium loading, chromium speciation, chemical/mineralogical residue composition, leaching resistance, or environmental containment.

At 3 and 6 wt.% residue, the processed binder and mortar master curves showed no large descriptive shifts relative to the processed control within the available specimen set. The 6 wt.% binder showed lower penetration, and the adapted nontandard mortar‐cylinder cyclic recovery metric increased from 87.3% for the control to 92.5% for 6 wt.% residue. Because binder batches were not independently replicated and only two mortar specimens were tested per condition, these results are descriptive compatibility signals rather than statistically demonstrated performance improvements.

Before practical reuse or treatment implementation can be considered, future work should include real tannery wastewater, independent replication, broader pH and competing‐ion conditions, total chromium and Cr (VI)/Cr (III) speciation, direct characterization of the recovered residue, ${\mathrm{pH}}_{\mathrm{pzc}}$/zeta‐potential and surface analyses where mechanistic interpretation is intended, regeneration efficiency and adsorbent stability across repeated adsorption–desorption cycles, leaching under water contact and wet–dry cycling, long‐term aging, and standardized full‐asphalt‐mixture performance testing.

## Author Contributions

**Kevin Yonny Avila‐Robayo:** investigation, methodology, formal analysis, data curation. **Eduardo J. Rueda:** conceptualization, writing – original draft, supervision. **Jaime Plazas‐Tuttle:** conceptualization, methodology, data curation, writing – original draft, writing – review and editing, visualization, formal analysis, supervision, validation.

## Funding

The authors have nothing to report.

## Conflicts of Interest

The authors declare no conflicts of interest.

## Declaration of Generative AI and AI‐Assisted Technologies in the Writing Process

During manuscript preparation, generative AI tools were used to support language editing, manuscript organization, and refinement of scientific wording. These tools were not used to generate experimental data, create results, perform unverified analyses, or make autonomous scientific conclusions. All AI‐assisted text was reviewed, edited, and approved by the authors, who remain fully responsible for the content of the manuscript.

## Supporting information




**Table S1:** 1 Materials, reagents, and major equipment used in the experimental workflow.
**Table S2:** 1. Operating conditions for bone char preparation.
**Table S2:** 3. Milling and dispersion sequence used for bone char particle size conditioning.
**Table S3:** 1. Cr (VI) stock solution, intermediate solution, color‐development conditions, and analytical‐range notes.
**Table S3:** 2. Dilution volumes for Cr (VI) calibration standards.
**Table S3:** 3. Documented dilution information and sample‐equivalent lower calibration concentrations for adsorption supernatants.
**Table S4:** 1. Sample preparation volumes for the contact‐time screening assay.
**Table S4:** 2. Raw contact‐time screening results, including time‐matched process blanks.
**Table S4:** 3. Sample preparation volumes for the initial 120‐min dose–response screening assay.
**Table S4:** 4. Sample preparation volumes for the refined 120‐min dose–response assay.
**Table S4:** 5. Initial 120‐min dose–response screening assay results.
**Table S4:** 6. Refined 120‐min dose–response dataset used for nonlinear isotherm model fitting.
**Table S4:** 7. Sensitivity of apparent removal to the time‐matched process blank in the contact‐time screening assay.
**Table S5:** 1. Nonlinear isotherm‐model parameters and comparison metrics for the refined 120‐min dose–response dataset.
**Table S5:** 2. Observed and nonlinear model‐predicted apparent uptake values for the refined 120‐min dose–response dataset.
**Table S6:** 1. Preparation conditions for the process‐derived BC/Cr residue used in asphalt‐compatible processing tests.
**Table S6:** 2. Moisture and recovered‐mass support for the process‐derived BC/Cr residue.
**Table S6:** 3. Rehydration and filtration indicators for water‐soluble material in the process‐derived residue.
**Table S6:** 4. Preparation replicas and acid/dichromate control observations.
**Table S7:** 1. Asphalt binder residue masses and blending conditions.
**Table S7:** 2. Binder blending sequence.
**Table S7:** 3. Individual penetration readings for asphalt binders.
**Table S7:** 4. Binder WLF coefficients and experimentally selected shift factors.
**Table S7:** 5. Binder replication structure and interpretation limit.
**Table S8:** 1. Asphalt mortar mixture proportioning and specimen preparation conditions.
**Table S8:** 2. Aggregate gradation used for asphalt mortar preparation.
**Table S9:** 1. Mortar WLF coefficients and experimentally selected shift factors.
**Table S9:** 2. Adapted nonstandard mortar‐cylinder cyclic creep–recovery testing conditions.
**Table S9:** 3. Cycle‐by‐cycle recovery percentages for control asphalt mortar.
**Table S9:** 4. Cycle‐by‐cycle recovery percentages for asphalt mortar prepared with 3 wt.% process‐derived residue.
**Table S9:** 5. Cycle‐by‐cycle recovery percentages for asphalt mortar prepared with 6 wt.% process‐derived residue.
**Table S9:** 6. Mortar replication structure and interpretation limit.
**Table S9:** 7. Specimen‐level and condition‐level cyclic recovery summary supporting Figure 7c.
**Table S10:** 1. Additional validation needs before environmental or practical implementation claims.

## Data Availability

All data available for the analyses reported in this study are contained within the article and its Supporting Information. The analyses reported in the manuscript were performed using these archived tabulated data; no additional raw instrument files or experimental datasets are available.
