# Supplementary material for: Waste‐Derived Fine‐Milled Bone Char for Apparent Cr (VI) Removal From Water and Preliminary Asphalt‐Compatible Processing of the Recovered Residue
Source: Water Environ Res. 2026 Sep 17;98(9):e70580. doi: 10.1002/wer.70580 (PMC13584846; doi:10.1002/wer.70580)
Supplement: Supplementary file 1 [file WER-98-e70580-s001.docx]

**Supplementary Information**

**Waste-Derived Fine-Milled Bone Char for Apparent Cr(VI) Removal from Water and Preliminary Asphalt-Compatible Processing of the Recovered Residue**

Kevin Yonny Avila-Robayo¹, Eduardo J. Rueda-Cárdenas², Jaime Plazas-Tuttle¹,³,*

*¹Department of Civil and Environmental Engineering, Universidad de los Andes, Bogotá, Colombia*

*²Escuela de Construcción Civil, Facultad de Ingeniería, Pontificia Universidad Católica de Chile, Santiago, Chile*

*³Water Distribution and Sewerage Systems Research Center (CIACUA), Universidad de los Andes, Bogotá, Colombia*

*Corresponding author. Tel: +57-1-339-4949. E-mail: jplazas@uniandes.edu.co (Jaime Plazas-Tuttle)

E-mail addresses: ky.avila@uniandes.edu.co (Kevin Yonny Avila-Robayo), eduardo.rueda@uc.cl (Eduardo J. Rueda-Cárdenas), jplazas@uniandes.edu.co (Jaime Plazas-Tuttle).

# Scope and non-redundancy principle

This Supplementary Information provides protocol-level details, sample-preparation records, archived tabulated datasets, and supporting calculations used in the revised article. It documents the analytical calibration and dilution records, including the unresolved sample-equivalent lower calibration bound for the contact-time assay; the time-matched blank sensitivity calculation; nonlinear isotherm-model fitting of the refined 120 min dose-response dataset; the compositionally unresolved process-derived residue; rheological replication limits; the adapted non-standard mortar-cylinder cyclic recovery calculations; and validation needs. Article-level summary figures are not duplicated; tabulated support is provided where additional auditability is useful.

# Contents

- S1. Materials, reagents, equipment, and operating conditions
- S2. Bone-char preparation and milling support
- S3. Cr(VI) quantification, calibration range, and dilution records
- S4. Adsorption sample preparation records and raw adsorption data
- S5. Nonlinear isotherm-model fitting support
- S6. Process-derived BC/Cr residue preparation and composition support
- S7. Asphalt binder modification, penetration readings, and DSR support
- S8. Asphalt mortar mixture design and specimen preparation support
- S9. Asphalt mortar rheology, adapted non-standard cyclic recovery data, and replication support
- S10. Interpretation boundary and additional validation matrix

# S1. Materials, reagents, equipment, and operating conditions

Table S1.1. Materials, reagents, and major equipment used in the experimental workflow.

| **Item** | **Use** | **Specification or note** |
| --- | --- | --- |
| Bovine femur bone | Precursor for bone char production | Obtained from a slaughterhouse near Bogotá, Colombia |
| Virgin 60-70 penetration-grade asphalt binder | Asphalt binder modification and mortar preparation | Supplied by the Civil Engineering Laboratory, Universidad de los Andes |
| Potassium dichromate (K2Cr2O7) | Cr(VI) stock solution | Sigma-Aldrich/Merck, ACS reagent grade, >=99.0% |
| Sulfuric acid (H2SO4) | pH adjustment and acidic Cr(VI) conditions | Sigma-Aldrich/Merck, ACS reagent grade, 95.0-98.0% |
| 1,5-diphenylcarbazide in acetone | Colorimetric Cr(VI) quantification | Prepared fresh/stored protected from light |
| Distilled water | Solution preparation, dilution, suspension preparation, and washing | Used throughout adsorption procedures |
| High-temperature furnace under nitrogen | Thermal conversion of bone precursor | Nitrogen atmosphere; 700-800 deg C holding interval |
| High-energy roller mill | First particle-size reduction stage | Seven 30 s milling cycles |
| Planetary ball mill | Second particle-size reduction stage | Three 1.5 h cycles with 7.0 or 9.5 g per vessel |
| Laser-diffraction particle-size analyzer | Particle-size distribution measurement | Measurements after sonication-assisted dispersion |
| UV-Vis spectrophotometer | Cr(VI) concentration measurement | 540 nm, 5 cm optical path-length glass cells |
| Tube rotator and vortex mixer | Batch adsorption contact | Vortex mixing for 30 s before rotation |
| Centrifuge | Solid-liquid separation after contact | 4000 rpm for 5 min |
| Dynamic shear rheometer (DSR) | Binder and mortar rheological characterization | Parallel plates for binders; clamping system for cylindrical mortars |
| Asphalt penetrometer | Binder consistency | 25 deg C penetration test with three readings per condition |

# S2. Bone-char preparation and milling support

Table S2.1. Operating conditions for bone-char preparation.

| **Parameter** | **Condition** |
| --- | --- |
| Selected bone section | Central portion of bovine femur |
| Manual cleaning | Removal of external tissue, marrow, and internal organic residues |
| Water-bath pretreatment | 80 deg C for 3 h |
| Drying before crushing | 103 deg C for 2 h |
| Crushed bone introduced into furnace | 100.12 g |
| Thermal atmosphere | Nitrogen |
| Temperature ramp | Approximately 5 h |
| Holding interval | 700-800 deg C for 2 h |
| Operating pressure | Approximately 10 psi |
| Solid recovered after thermal conversion | 68.96 g |
| Sequentially milled material available for experiments | 52 g |
| Chemical activation or surface functionalization | Not applied |

Table S2.2. Bone char production yield and particle-size metrics after sequential milling and sonication-assisted laser diffraction.

| **Parameter** | **BC-MA** | **BC-MB** |
| --- | --- | --- |
| Milling condition | High-energy roller milling | High-energy roller milling + planetary ball milling |
| D10 (µm) | 1.13 | 0.78 |
| D50 (µm) | 9.72 | 5.52 |
| D90 (µm) | 42.51 | 23.32 |
| Mean particle diameter (µm) | 16.13 | 9.08 |
| Use in this study | Particle-size characterization only | Adsorption and asphalt-compatible incorporation experiments |

Table S2.3. Milling and dispersion sequence used for bone-char particle-size conditioning.

| **Material or step** | **Operation** | **Detailed condition** | **Role in study** |
| --- | --- | --- | --- |
| BC-MA | High-energy roller milling | Seven cycles of 30 s each | Characterized for comparison |
| BC-MB | Roller milling plus planetary ball milling | One 1.5 h cycle with 7.0 g per vessel; two additional 1.5 h cycles with 9.5 g per vessel | Selected for adsorption and asphalt incorporation |
| Dispersion before particle-size analysis | Sonication-assisted aqueous dispersion | Additional sonication applied before laser diffraction | Used to reduce agglomeration before measurement |

Particle-size percentiles are reported to characterize the BC-MB fraction as fine-milled BC with a submicron fraction (D50 = 5.52 µm; D10 = 0.78 µm). The revised article therefore uses “fine-milled BC with a submicron fraction” rather than “ultrafine” or “nanoparticle” terminology.

# S3. Cr(VI) quantification, calibration range, and dilution records

Cr(VI) was quantified using the acidic 1,5-diphenylcarbazide colorimetric method. Calibration standards covered 0.00–0.40 mg L⁻¹ Cr(VI), with 0.020 mg L⁻¹ as the lowest non-zero standard in the analytical flask. Archived dose-response records document a 5-fold dilution for the nominal 2 mg L⁻¹ series and a 50-fold dilution for the nominal 20 mg L⁻¹ series; the 0.020 mg L⁻¹ analytical standard therefore corresponds to sample-equivalent concentrations of 0.10 and 1.0 mg L⁻¹, respectively, before dilution. For the contact-time assay, the archived procedure confirms dilution of the supernatant to 50 mL but does not record the aliquot volume, so its sample-equivalent lower calibration bound cannot be reconstructed. The records do not contain replicate blank/calibration statistics sufficient to establish formal method detection (LOD) or quantification (LOQ) limits. Very low archived concentrations are therefore retained for transparency but are interpreted qualitatively whenever they fall below a documented sample-equivalent calibration range or when that range cannot be reconstructed.

Table S3.1. Cr(VI) stock solution, intermediate solution, color-development conditions, and analytical-range notes.

| **Item** | **Preparation or condition** |
| --- | --- |
| Cr(VI) stock solution | 565.78 mg K2Cr2O7 diluted to 500 mL; pH adjusted below 2 |
| Nominal stock concentration | 0.4 mg Cr(VI) mL-1 |
| Intermediate Cr(VI) solution | 1 mL stock solution diluted to 100 mL |
| Nominal intermediate concentration | 0.004 mg Cr(VI) mL-1 |
| Colorimetric reagent | 250 mg 1,5-diphenylcarbazide dissolved in 50 mL acetone |
| Color development | 1.0 mL reagent added to 50 mL flask; 5-10 min before reading |
| Measurement wavelength | 540 nm |
| Optical path length | 5 cm |
| Calibration acceptance check | One standard verified within +/-10% before proceeding when the curve was reused |
| Lowest non-zero calibration standard | 0.020 mg L⁻¹ Cr(VI) in the analytical flask |
| Formal LOD/LOQ | Not determined from replicate blank/calibration statistics; interpretation uses the documented calibration range and sample-specific dilution information |
| Sample-equivalent lower calibration concentration, 2 mg L⁻¹ dose-response series | 0.10 mg L⁻¹ before dilution (5-fold dilution) |
| Sample-equivalent lower calibration concentration, 20 mg L⁻¹ dose-response series | 1.0 mg L⁻¹ before dilution (50-fold dilution) |
| Contact-time assay sample-equivalent lower calibration concentration | Not reconstructable from archived records because the aliquot volume before dilution to 50 mL was not recorded |

Table S3.2. Dilution volumes for Cr(VI) calibration standards.

| **Standard** | **Final volume (mL)** | **Intermediate Cr(VI) solution volume (mL)** | **Cr(VI) concentration (mg/L)** |
| --- | --- | --- | --- |
| Blank | 50 | 0.0000 | 0.0000 |
| C-1 | 50 | 0.2500 | 0.0200 |
| C-2 | 50 | 0.5000 | 0.0400 |
| C-3 | 50 | 1.25 | 0.1000 |
| C-4 | 50 | 2.50 | 0.2000 |
| C-5 | 50 | 3.75 | 0.3000 |
| C-6 | 50 | 5.00 | 0.4000 |

Table S3.3. Documented dilution information and sample-equivalent lower calibration concentrations for adsorption supernatants.

| **Assay / nominal sample range** | **Aliquot (mL)** | **Final volume (mL)** | **Dilution factor** | **Analytical concentration if no removal** | **Sample-equivalent lowest non-zero calibration concentration** |
| --- | --- | --- | --- | --- | --- |
| Dose-response, 2 mg L⁻¹ Cr(VI) | 10 | 50 | 5× | 0.40 mg L⁻¹ | 0.10 mg L⁻¹ |
| Dose-response, 20 mg L⁻¹ Cr(VI) | 1 | 50 | 50× | 0.40 mg L⁻¹ | 1.0 mg L⁻¹ |
| Contact-time screening, nominal 2 mg L⁻¹ Cr(VI) | Not recorded | 50 | Not reconstructable | Not reconstructable | Not reconstructable |

# S4. Adsorption sample preparation records and raw adsorption data

Table S4.1. Sample preparation volumes for the contact-time screening assay.

| **Sample** | **Final volume (mL)** | **BC solution volume (mL)** | **BC dose (g/L)** | **Cr(VI) solution volume (mL)** | **Initial Cr(VI) (mg/L)** | **Distilled water (mL)** |
| --- | --- | --- | --- | --- | --- | --- |
| Blank T1 | 15 | 0 | 0 | 0.0750 | 2 | 14.93 |
| T-1 | 15 | 7.50 | 1 | 0.0750 | 2 | 7.425 |
| Blank T2 | 15 | 0 | 0 | 0.0750 | 2 | 14.93 |
| T-2 | 15 | 7.50 | 1 | 0.0750 | 2 | 7.425 |
| Blank T3 | 15 | 0 | 0 | 0.0750 | 2 | 14.93 |
| T-3 | 15 | 7.50 | 1 | 0.0750 | 2 | 7.425 |
| Blank T4 | 15 | 0 | 0 | 0.0750 | 2 | 14.93 |
| T-4 | 15 | 7.50 | 1 | 0.0750 | 2 | 7.425 |
| Blank T5 | 15 | 0 | 0 | 0.0750 | 2 | 14.93 |
| T-5 | 15 | 7.50 | 1 | 0.0750 | 2 | 7.425 |

Table S4.2. Raw contact-time screening results, including time-matched process blanks.

| **Sample** | **BC dose (g/L)** | **Initial Cr(VI) (mg/L)** | **Contact time (h)** | **Final Cr(VI), Ce (mg/L)** | **Removal (%)** | **qe (mg/g)** |
| --- | --- | --- | --- | --- | --- | --- |
| Blank T1 | 0 | 2 | 0.5000 | 1.98 | 0.8200 | 0 |
| Blank T2 | 0 | 2 | 1.00 | 1.95 | 2.49 | 0 |
| Blank T3 | 0 | 2 | 2.00 | 1.86 | 7.24 | 0 |
| Blank T4 | 0 | 2 | 3.00 | 2.01 | -0.2700 | 0 |
| Blank T5 | 0 | 2 | 4.00 | 2.09 | -4.44 | 0 |
| T-1 | 1 | 2 | 0.5000 | 0.0600 | 97.24 | 1.94 |
| T-2 | 1 | 2 | 1.00 | 0.0300 | 98.52 | 1.97 |
| T-3 | 1 | 2 | 2.00 | 0.0200 | 98.78 | 1.98 |
| T-4 | 1 | 2 | 3.00 | 0.0300 | 98.52 | 1.97 |
| T-5 | 1 | 2 | 4.00 | 0.0400 | 97.95 | 1.96 |

Note. One BC-containing sample and one process blank were analyzed at each contact time. The experiment was designed to select a standardized operational contact time, not to support kinetic-model inference. The archived procedure does not document the aliquot volume used before dilution to 50 mL; therefore, a sample-equivalent lower calibration bound cannot be reconstructed for this assay. The very low residual concentrations are retained as archived measurements but small differences among later contact times are not interpreted quantitatively. qₑ is calculated from aqueous Cr(VI) depletion and therefore represents apparent uptake rather than direct measurement of chromium retained in the solid phase.

Table S4.3. Sample preparation volumes for the initial 120 min dose-response screening assay.

| **Sample** | **Final volume (mL)** | **BC solution volume (mL)** | **BC dose (g/L)** | **Cr(VI) solution volume (mL)** | **Initial Cr(VI) (mg/L)** | **Distilled water (mL)** |
| --- | --- | --- | --- | --- | --- | --- |
| Blank A | 15 | 0 | 0 | 7.50 | 2 | 7.50 |
| A-1 | 15 | 0.7500 | 1 | 7.50 | 2 | 6.75 |
| A-2 | 15 | 1.50 | 2 | 7.50 | 2 | 6 |
| A-3 | 15 | 3.75 | 5 | 7.50 | 2 | 3.75 |
| A-4 | 15 | 7.50 | 10 | 7.50 | 2 | 0 |
| Blank A2 | 15 | 0 | 0 | 0.7500 | 20 | 14.25 |
| A-5 | 15 | 0.7500 | 1 | 0.7500 | 20 | 13.50 |
| A-6 | 15 | 1.50 | 2 | 0.7500 | 20 | 12.75 |
| A-7 | 15 | 3.75 | 5 | 0.7500 | 20 | 10.50 |
| A-8 | 15 | 7.50 | 10 | 0.7500 | 20 | 6.75 |

Table S4.4. Sample preparation volumes for the refined 120 min dose-response assay.

| **Sample** | **Final volume (mL)** | **BC solution volume (mL)** | **BC dose (g/L)** | **Cr(VI) solution volume (mL)** | **Initial Cr(VI) (mg/L)** | **Distilled water (mL)** |
| --- | --- | --- | --- | --- | --- | --- |
| Blank C | 15 | 0.0000 | 0.0000 | 0.7500 | 2 | 14.25 |
| C-1 | 15 | 0.5000 | 0.1000 | 0.7500 | 2 | 13.75 |
| C-2 | 15 | 1.00 | 0.2000 | 0.7500 | 2 | 13.25 |
| C-3 | 15 | 2.50 | 0.5000 | 0.7500 | 2 | 11.75 |
| C-4 | 15 | 3.50 | 0.7000 | 0.7500 | 2 | 10.75 |
| C-5 | 15 | 5.00 | 1.00 | 0.7500 | 2 | 9.25 |
| Blank C2 | 15 | 0.0000 | 0.0000 | 7.50 | 20 | 7.50 |
| C-6 | 15 | 0.5000 | 1.00 | 7.50 | 20 | 7.00 |
| C-7 | 15 | 1.00 | 2.00 | 7.50 | 20 | 6.50 |
| C-8 | 15 | 2.50 | 5.00 | 7.50 | 20 | 5.00 |
| C-9 | 15 | 3.50 | 7.00 | 7.50 | 20 | 4.00 |
| C-10 | 15 | 5.00 | 10.00 | 7.50 | 20 | 2.50 |

Table S4.5. Initial 120 min dose-response screening assay results.

| **Sample** | **BC dose (g/L)** | **Initial Cr(VI) (mg/L)** | **Contact time (h)** | **Ce (mg/L)** | **Removal (%)** | **qe (mg/g)** |
| --- | --- | --- | --- | --- | --- | --- |
| Blank A | 0 | 2 | 2 | 1.94 | 3.20 | 0 |
| A-1 | 1 | 2 | 2 | 0.0200 | 99.10 | 1.98 |
| A-2 | 2 | 2 | 2 | 0.0200 | 99.10 | 0.9900 |
| A-3 | 5 | 2 | 2 | 0.0400 | 98.08 | 0.3900 |
| A-4 | 10 | 2 | 2 | 0.0100 | 99.68 | 0.2000 |
| Blank A2 | 0 | 20 | 2 | 19.82 | 0.8900 | 0 |
| A-5 | 1 | 20 | 2 | 15.61 | 21.93 | 4.39 |
| A-6 | 2 | 20 | 2 | 12.43 | 37.84 | 3.78 |
| A-7 | 5 | 20 | 2 | 6.68 | 66.58 | 2.66 |
| A-8 | 10 | 20 | 2 | 6.81 | 65.94 | 1.32 |

Note. The screening assay documents near-complete apparent Cr(VI) depletion under selected low-concentration conditions. For the nominal 2 mg L⁻¹ series, the documented 5-fold dilution gives a sample-equivalent lowest non-zero calibration concentration of 0.10 mg L⁻¹; archived residual values below 0.10 mg L⁻¹ are therefore retained for transparency but interpreted qualitatively. The highest observed apparent qₑ, 4.39 mg g⁻¹, occurred in the 20 mg L⁻¹ series, for which the corresponding diluted analytical concentration remained within the calibration range. The screening dataset was not combined with the refined dataset for nonlinear model fitting.

Table S4.6. Refined 120 min dose-response dataset used for nonlinear isotherm-model fitting.

| **Sample** | **C₀ (mg L⁻¹)** | **BC dose (g L⁻¹)** | **Cₑ at 120 min (mg L⁻¹)** | **Apparent removal (%)** | **Apparent qₑ (mg g⁻¹)** |
| --- | --- | --- | --- | --- | --- |
| Blank C | 2 | 0.0000 | 1.95 | 2.36 | 0.0000 |
| C-1 | 2 | 0.1000 | 1.87 | 6.41 | 1.28 |
| C-2 | 2 | 0.2000 | 1.70 | 14.81 | 1.48 |
| C-3 | 2 | 0.5000 | 1.35 | 32.71 | 1.31 |
| C-4 | 2 | 0.7000 | 1.15 | 42.59 | 1.22 |
| C-5 | 2 | 1.00 | 0.8800 | 55.93 | 1.12 |
| Blank C2 | 20 | 0.0000 | 19.71 | 1.47 | 0.0000 |
| C-6 | 20 | 1.00 | 17.65 | 11.73 | 2.35 |
| C-7 | 20 | 2.00 | 15.58 | 22.12 | 2.21 |
| C-8 | 20 | 5.00 | 9.89 | 50.54 | 2.02 |
| C-9 | 20 | 7.00 | 6.38 | 68.12 | 1.95 |
| C-10 | 20 | 10.00 | 3.04 | 84.80 | 1.70 |

*Note. All adsorbent-containing residual concentrations in this refined dataset fall within the non-zero analytical calibration range after applying the documented dilution factors. Each dose condition was represented by one independently prepared batch tube (n = 1); the dataset is therefore interpreted descriptively and was not used for inferential statistical testing.*

Table S4.7. Sensitivity of apparent removal to the time-matched process blank in the contact-time screening assay.

| **Contact time (min)** | **Time-matched blank Cr(VI) (mg L⁻¹)** | **BC-containing sample Cr(VI) (mg L⁻¹)** | **Blank-referenced apparent removal (%)** |
| --- | --- | --- | --- |
| 30 | 1.98 | 0.06 | 96.97 |
| 60 | 1.95 | 0.03 | 98.46 |
| 120 | 1.86 | 0.02 | 98.92 |
| 180 | 2.01 | 0.03 | 98.51 |
| 240 | 2.09 | 0.04 | 98.09 |

Note. The time-matched blanks vary from 1.86 to 2.09 mg L⁻¹. Using each blank as the local reference still gives approximately 97–99% calculated apparent Cr(VI) depletion in the corresponding BC-containing samples, so the observed decrease is substantially greater than the blank variability. However, the absence of independent replicates and the unresolved sample-equivalent lower calibration bound for the contact-time assay mean that small differences among the later low residual concentrations should not be interpreted quantitatively.

# S5. Nonlinear isotherm-model fitting support

The ten adsorbent-containing observations in the refined 120 min dose-response dataset (Table S4.6) were fitted directly in qₑ–Cₑ space by unweighted nonlinear least squares. Langmuir, Freundlich, Sips, and Redlich–Peterson isotherm models were compared using R², RMSE, and the small-sample corrected Akaike information criterion (AICc). For consistency with conventional isotherm notation, Cₑ and qₑ denote the residual concentration and calculated apparent aqueous-depletion-based uptake at the standardized 120 min endpoint; the subscript e does not imply that equilibrium was independently demonstrated. The fitted models are used only as empirical descriptors of the observed 120 min qₑ–Cₑ relationship and not as validated equilibrium design relationships or evidence of a specific removal mechanism.

Table S5.1. Nonlinear isotherm-model parameters and comparison metrics for the refined 120 min dose-response dataset.

| **Model** | **Nonlinear fitted parameters** | **R²** | **RMSE (mg g⁻¹)** | **AICc** |
| --- | --- | --- | --- | --- |
| Langmuir | qmax = 2.355 mg g⁻¹; KL = 0.884 L mg⁻¹ | 0.955 | 0.0893 | −42.59 |
| Freundlich | KF = 1.223 (mg g⁻¹)(L mg⁻¹)^(1/n); n = 4.423 | 0.964 | 0.0795 | −44.93 |
| Sips | qs = 3.087 mg g⁻¹; Ks = 0.403 L mg⁻¹; ns = 0.536 | 0.974 | 0.0682 | −43.69 |
| Redlich–Peterson | KR = 4.318 L g⁻¹; aR = 2.696 (L mg⁻¹)^β; β = 0.864 | 0.975 | 0.0673 | −43.96 |

Table S5.2. Observed and nonlinear-model-predicted apparent uptake values for the refined 120 min dose-response dataset.

| **Sample** | **Cₑ (mg L⁻¹)** | **Observed qₑ (mg g⁻¹)** | **Langmuir qₑ** | **Freundlich qₑ** | **Sips qₑ** | **Redlich–Peterson qₑ** |
| --- | --- | --- | --- | --- | --- | --- |
| C-1 | 1.87 | 1.28 | 1.467 | 1.409 | 1.427 | 1.434 |
| C-2 | 1.70 | 1.48 | 1.414 | 1.379 | 1.387 | 1.394 |
| C-3 | 1.35 | 1.31 | 1.281 | 1.309 | 1.294 | 1.297 |
| C-4 | 1.15 | 1.22 | 1.187 | 1.262 | 1.230 | 1.228 |
| C-5 | 0.88 | 1.12 | 1.030 | 1.188 | 1.125 | 1.113 |
| C-6 | 17.65 | 2.35 | 2.213 | 2.340 | 2.288 | 2.295 |
| C-7 | 15.58 | 2.21 | 2.195 | 2.275 | 2.247 | 2.249 |
| C-8 | 9.89 | 2.02 | 2.113 | 2.053 | 2.090 | 2.081 |
| C-9 | 6.38 | 1.95 | 2.000 | 1.859 | 1.926 | 1.917 |
| C-10 | 3.04 | 1.70 | 1.716 | 1.573 | 1.627 | 1.631 |

*Note. Predicted qₑ values are shown to support auditability of Figure 4 and the model-comparison metrics in Table S5.1. Values are calculated from the nonlinear fitted parameters; minor differences from article display values can result from rounding.*

Note. Sips and Redlich–Peterson marginally reduce residual error relative to the two-parameter models, but the additional parameter increases uncertainty in this small dataset. Freundlich provides the lowest AICc because of its parsimony. Because equilibrium was not independently demonstrated, no fitted model is treated as a validated equilibrium relationship or as evidence of a specific surface mechanism; qmax and qs are fitted capacity parameters rather than experimentally demonstrated maximum adsorption capacities.

# S6. Process-derived BC/Cr residue preparation and composition support

The asphalt-compatible processing tests used the entire dried residue produced after acidic Cr(VI)/dichromate contact and evaporative drying. Because chromium retained in the solid was not directly measured and a substantial unresolved additional dry mass was recovered, this material is designated the process-derived BC/Cr residue rather than a measured “Cr-loaded BC” material. The 1.6 mg Cr(VI) g⁻¹ value is a nominal input ratio only (64 mg Cr(VI) added / 40.0043 g initial BC); it is not a measured solid-phase chromium loading or a fraction of an adsorption capacity.

Table S6.1. Preparation conditions for the process-derived BC/Cr residue used in asphalt-compatible processing tests.

| **Parameter** | **Value or condition** |
| --- | --- |
| BC fraction | BC-MB |
| BC mass | 40.0043 g |
| Initial suspension volume | 500 mL distilled water |
| Sonication before contact | 3 min |
| Cr(VI) stock solution added | 160 mL |
| Nominal Cr(VI) mass added | 64 mg |
| Nominal Cr(VI) input ratio | 1.6 mg Cr(VI) added g⁻¹ initial BC; retained chromium not measured |
| Final suspension volume | 750 mL |
| pH adjustment | Concentrated H2SO4 to pH < 2 |
| Contact time before drying | 2 h under magnetic stirring (operational contact time) |
| Drying condition | 50 deg C until complete evaporation |
| Approximate drying time | 11 days |
| Post-drying treatment | Manual disaggregation, maceration, and storage |

Table S6.2. Moisture and recovered-mass support for the process-derived BC/Cr residue.

| **Measurement or calculation** | **Value** |
| --- | --- |
| Recovered process-derived residue mass after drying | 68.95 g |
| Initial subsample for moisture test | 10.1002 g |
| Dry subsample mass after 103 deg C drying | 9.1869 g |
| Moisture content | 9.04% |
| Estimated dry mass of recovered process-derived residue | 62.72 g |
| Estimated unresolved additional dry material | 22.65 g |

Table S6.3. Rehydration and filtration indicators for water-soluble material in the process-derived residue.

| **Condition** | **Initial residue mass (g)** | **Initial dry mass (g)** | **Unresolved additional dry material (g)** | **Final filtered mass (g)** | **Soluble mass (g)** | **Soluble/initial dry mass (%)** | **Soluble/unresolved additional dry material (%)** |
| --- | --- | --- | --- | --- | --- | --- | --- |
| Room temperature | 5.002 | 4.55 | 1.642 | 3.318 | 1.232 | 27.07 | 74.98 |
| 50 deg C | 5.001 | 4.549 | 1.642 | 3.232 | 1.327 | 29.17 | 80.82 |
| Scaled replica, room temperature | 4.71 | 4.71 | 1.711 | 3.318 | 1.391 | 29.54 | 81.34 |

Table S6.4. Preparation replicas and acid/dichromate control observations.

| **Trial** | **Preparation basis** | **Observed outcome** |
| --- | --- | --- |
| Original preparation | 40.0043 g BC; 160 mL Cr(VI) stock; pH adjusted below 2; dried at 50 deg C | 68.95 g recovered material |
| Scaled replica | 3.0 g BC; 12 mL Cr(VI) stock; 1 mL H2SO4; dried at 50 deg C | 4.71 g dried material |
| Acid/dichromate control without BC | 12 mL Cr(VI) stock; 1 mL H2SO4; no BC; dried first at 50 deg C and then at 103 deg C | 1.1 mL residual liquid; 1.9967 g |

# S7. Asphalt binder modification, penetration readings, and DSR support

Table S7.1. Asphalt binder residue masses and blending conditions.

| **Binder condition** | **Binder mass (g)** | **Process-derived residue dosage (wt.% binder)** | **Residue mass (g)** | **Binder temperature (deg C)** | **Mixing speed (rpm)** | **Mixing time (min)** |
| --- | --- | --- | --- | --- | --- | --- |
| Control binder | 75 | 0 | 0.0000 | 150-160 | 1250 | 40 |
| 3 wt.% residue binder | 75 | 3 | 2.25 | 150-160 | 1250 | 40 |
| 6 wt.% residue binder | 75 | 6 | 4.50 | 150-160 | 1250 | 40 |

Table S7.2. Binder blending sequence.

| **Step** | **Condition** |
| --- | --- |
| Initial heating of binder | 160 deg C for 30 min |
| Cooling before modification | 24 h to equalize thermal history |
| Reheating before blending | 160 deg C for 30 min |
| Mixing geometry | Mechanical stirrer positioned approximately at mid-depth |
| Modifier addition | Gradual addition to molten binder |
| Cooling after mixing | Samples cooled before penetration, rheology, and mortar preparation |

Table S7.3. Individual penetration readings for asphalt binders.

| **Binder condition** | **Reading 1 (0.1 mm)** | **Reading 2 (0.1 mm)** | **Reading 3 (0.1 mm)** | **Mean (0.1 mm)** |
| --- | --- | --- | --- | --- |
| Control binder | 50 | 52 | 52 | 51 |
| 3 wt.% process-derived residue | 50 | 52 | 51 | 51 |
| 6 wt.% process-derived residue | 47 | 46 | 43 | 45 |

Table S7.4. Binder WLF coefficients and experimentally selected shift factors.

| **Binder** | **Specimen** | **C₁** | **C₂ (°C)** | **a_T, 25 °C** | **a_T, 35 °C** | **a_T, 45 °C** | **a_T, 55 °C** | **a_T, 65 °C** |
| --- | --- | --- | --- | --- | --- | --- | --- | --- |
| Control | M1 | 11.42 | 100.38 | 1.00 | 0.1000 | 0.0120 | 0.0022 | 0.0006 |
| Control | M2 | 11.86 | 102.46 | 1.00 | 0.1000 | 0.0100 | 0.0015 | 0.0003 |
| Control | M3 | 10.22 | 91.87 | 1.00 | 0.1000 | 0.0150 | 0.0030 | 0.0010 |
| 3 wt.% | M1 | 11.96 | 101.96 | 1.00 | 0.1000 | 0.0150 | 0.0020 | 0.0005 |
| 3 wt.% | M2 | 8.30 | 69.45 | 1.00 | 0.1000 | 0.0170 | 0.0045 | 0.0015 |
| 3 wt.% | M3 | 10.22 | 91.88 | 1.00 | 0.1000 | 0.0150 | 0.0030 | 0.0010 |
| 6 wt.% | M1 | 10.22 | 91.88 | 1.00 | 0.1000 | 0.0150 | 0.0030 | 0.0010 |
| 6 wt.% | M2 | 10.22 | 91.88 | 1.00 | 0.1000 | 0.0150 | 0.0030 | 0.0010 |
| 6 wt.% | M3 | 8.75 | 74.94 | 1.00 | 0.1000 | 0.0150 | 0.0040 | 0.0010 |

*Note. Tref = 25 °C. a_T is dimensionless; C₁ is dimensionless; C₂ has units of °C. The 75 °C binder data were excluded from article master-curve construction because unreliable values were observed at that temperature.*

Note. The 75 °C binder data were not used in article master-curve construction because unreliable values were observed at this temperature.

Table S7.5. Binder replication structure and interpretation limit.

| **Item** | **Count / status** | **Interpretation** |
| --- | --- | --- |
| Processed binder batches per condition | 1 | Control, 3 wt.%, and 6 wt.% were each prepared as one 75 g processed batch |
| DSR specimens per condition | 3 | Three rheometer subsamples from the corresponding single processed binder batch; not independent formulation-level preparation replicates |
| Inferential comparison | Not performed in revision | Frequency points and within-batch specimens do not provide independent preparation replication |

Note. Frequency points within a master curve are repeated measurements and are not independent experimental units. The revised manuscript therefore does not report ANOVA p-values for binder master curves.

# S8. Asphalt mortar mixture design and specimen preparation support

Table S8.1. Asphalt mortar mixture proportioning and specimen preparation conditions.

| **Parameter** | **Value or condition** |
| --- | --- |
| Target binder content | 9.75% by total mixture mass |
| Target air-void content | 10% |
| Design bulk specific gravity, Gmb | 2.01 |
| Mixture prepared per specimen | 20 g |
| Mass placed into cylindrical mold | 14.84 g |
| Mold internal diameter | 1.391 cm |
| Mold height | 4.86 cm |
| Conditioning before compaction | 140 deg C for 2 h |
| Compaction | Vertical air-compression system for 45 min |
| Specimen storage before testing | 24 h in desiccator; metallic supports bonded with epoxy; additional 24 h in desiccator |

*Note. The archived target binder content is 9.75% by total mixture mass; the main article reports this value as 9.8% after numerical rounding.*

Table S8.2. Aggregate gradation used for asphalt mortar preparation.

| **Sieve** | **Size (mm)** | **Retained (%)** | **Passing (%)** | **Mass (g)** | **Cumulative mass (g)** |
| --- | --- | --- | --- | --- | --- |
| No. 16 | 1.18 | 0 | 100 | 0.0000 | 0.0000 |
| No. 40 | 0.4200 | 35 | 65 | 6.32 | 6.32 |
| No. 80 | 0.1700 | 23 | 42 | 4.21 | 10.52 |
| No. 200 | 0.0750 | 28 | 13 | 5.11 | 15.63 |
| Pan | 0 | 13 | 0 | 2.42 | 18.05 |
| Total | - | 100 | - | 18.05 | - |

# S9. Asphalt mortar rheology, adapted non-standard cyclic recovery data, and replication support

Table S9.1. Mortar WLF coefficients and experimentally selected shift factors.

| **Mortar** | **Specimen** | **C₁** | **C₂ (°C)** | **a_T, 25 °C** | **a_T, 35 °C** | **a_T, 45 °C** | **a_T, 55 °C** | **a_T, 65 °C** | **a_T, 75 °C** |
| --- | --- | --- | --- | --- | --- | --- | --- | --- | --- |
| Control | M1 | 11.00 | 78.09 | 1.00 | 0.0700 | 0.0049 | 0.0008 | 0.0002 | 0.0001 |
| Control | M2 | 8.87 | 55.83 | 1.00 | 0.0600 | 0.0040 | 0.0006 | 0.0001 | 0.0001 |
| 3 wt.% | M1 | 10.80 | 74.35 | 1.00 | 0.0650 | 0.0040 | 0.0006 | 0.0001 | 0.0001 |
| 3 wt.% | M2 | 10.00 | 61.11 | 1.00 | 0.0600 | 0.0040 | 0.0006 | 0.0001 | 0.0001 |
| 6 wt.% | M1 | 9.74 | 74.00 | 1.00 | 0.0750 | 0.0090 | 0.0013 | 0.0003 | 0.0001 |
| 6 wt.% | M2 | 6.26 | 32.07 | 1.00 | 0.0750 | 0.0070 | 0.0009 | 0.0002 | 0.0001 |

*Note. Tref = 25 °C. a_T is dimensionless; C₁ is dimensionless; C₂ has units of °C. The 75 °C mortar data were retained because the mortar specimens showed stable behavior at that temperature.*

Table S9.2. Adapted non-standard mortar-cylinder cyclic creep–recovery testing conditions.

| **Parameter** | **Condition** |
| --- | --- |
| Mortar conditions | Control, 3 wt.% process-derived residue, and 6 wt.% process-derived residue |
| Specimens | Two cylindrical mortar specimens per condition; cycles are repeated measurements within each specimen |
| Test temperature | 40 deg C |
| Applied torsional stress | 10 kPa |
| Loading time per cycle | 1 s |
| Recovery time per cycle | 9 s |
| Number of cycles | 10 |
| Main response variable | Cycle-by-cycle recovery percentage |

Note. This procedure used solid cylindrical mortar specimens under torsional loading and was not the standard binder-film MSCR protocol. Results are therefore interpreted only as comparative, non-standard cyclic recovery data.

Table S9.3. Cycle-by-cycle recovery percentages for control asphalt mortar.

| **Cycle** | **Control M1 (%)** | **Control M2 (%)** | **Average control (%)** |
| --- | --- | --- | --- |
| 1 | 71.33 | 71.68 | 71.51 |
| 2 | 81.45 | 82.32 | 81.88 |
| 3 | 85.13 | 85.74 | 85.44 |
| 4 | 87.33 | 88.13 | 87.73 |
| 5 | 88.72 | 89.60 | 89.16 |
| 6 | 89.76 | 90.58 | 90.17 |
| 7 | 90.68 | 91.26 | 90.97 |
| 8 | 91.26 | 91.89 | 91.57 |
| 9 | 91.85 | 92.45 | 92.15 |
| 10 | 91.95 | 92.69 | 92.32 |
| Average | 86.95 | 87.63 | 87.29 |

Table S9.4. Cycle-by-cycle recovery percentages for asphalt mortar prepared with 3 wt.% process-derived residue.

| **Cycle** | **3 wt.% M1 (%)** | **3 wt.% M2 (%)** | **Average 3 wt.% (%)** |
| --- | --- | --- | --- |
| 1 | 76.90 | 69.20 | 73.05 |
| 2 | 86.63 | 83.14 | 84.89 |
| 3 | 89.81 | 86.80 | 88.31 |
| 4 | 91.68 | 88.90 | 90.29 |
| 5 | 92.89 | 90.13 | 91.51 |
| 6 | 93.69 | 91.30 | 92.49 |
| 7 | 94.28 | 92.03 | 93.16 |
| 8 | 94.75 | 92.57 | 93.66 |
| 9 | 95.21 | 92.99 | 94.10 |
| 10 | 95.26 | 93.14 | 94.20 |
| Average | 91.11 | 88.02 | 89.57 |

Table S9.5. Cycle-by-cycle recovery percentages for asphalt mortar prepared with 6 wt.% process-derived residue.

| **Cycle** | **6 wt.% M1 (%)** | **6 wt.% M2 (%)** | **Average 6 wt.% (%)** |
| --- | --- | --- | --- |
| 1 | 80.97 | 78.53 | 79.75 |
| 2 | 89.39 | 87.89 | 88.64 |
| 3 | 92.07 | 90.99 | 91.53 |
| 4 | 93.85 | 92.41 | 93.13 |
| 5 | 94.48 | 93.37 | 93.93 |
| 6 | 95.37 | 94.15 | 94.76 |
| 7 | 95.63 | 94.81 | 95.22 |
| 8 | 96.38 | 95.19 | 95.79 |
| 9 | 96.28 | 95.65 | 95.97 |
| 10 | 96.47 | 95.71 | 96.09 |
| Average | 93.09 | 91.87 | 92.48 |

Table S9.6. Mortar replication structure and interpretation limit.

| **Item** | **Count / status** | **Interpretation** |
| --- | --- | --- |
| Mortar specimens per condition | 2 | Two separately prepared cylindrical mortar specimens using binder from the single processed binder batch for that condition; limited specimen-level replication |
| Frequency/cycle observations | Repeated within specimen | Repeated measurements within each specimen; not independent experimental replicates |
| Inferential comparison | Not performed in revision | Results are reported descriptively because n = 2 does not support robust ANOVA/post-hoc inference |

Table S9.7. Specimen-level and condition-level cyclic recovery summary supporting Figure 7c.

| **Condition** | **Specimen M1 mean (%)** | **Specimen M2 mean (%)** | **Condition mean (%)** | **Between-specimen SD (%)** |
| --- | --- | --- | --- | --- |
| Control | 86.95 | 87.63 | 87.29 | 0.48 |
| 3 wt.% process-derived residue | 91.11 | 88.02 | 89.57 | 2.18 |
| 6 wt.% process-derived residue | 93.09 | 91.87 | 92.48 | 0.86 |

*Note. Between-specimen standard deviation is the sample standard deviation of the two specimen-level mean recovery values (n = 2). These values are descriptive and are not standard binder MSCR results.*

Recovery averaging procedure. For each mortar specimen, recovery was first averaged across the ten loading–recovery cycles. The two resulting specimen-level mean values were then averaged within each condition. The ten cycles are repeated measurements within a specimen and are not treated as independent replicates.

Note. Only two mortar specimens were available per condition. The revised manuscript therefore presents master curves and cyclic-recovery trends descriptively and does not report ANOVA p-values or post-hoc comparisons.

# S10. Interpretation boundary and additional validation matrix

Numerical precision was standardized in the revised article to avoid implying accuracy beyond the available measurements. Additional digits are retained in this Supplementary Information only when needed to reconstruct preparation volumes, mass balances, calibration levels, model fitting, or instrument-derived quantities. The notation Cₑ and qₑ is retained for conventional isotherm reporting, but in this study these symbols refer to the standardized 120 min endpoint and do not establish independently demonstrated equilibrium.

Table S10.1. Additional validation needs before environmental or practical implementation claims.

| **Validation need** | **Purpose** |
| --- | --- |
| Formal analytical LOD/LOQ and complete dilution/QA-QC records | Establish defensible lower reporting limits and quantify analytical uncertainty, particularly for very low residual Cr(VI) concentrations. |
| Independently replicated early-time contact study | Support defensible kinetic-model discrimination and independently demonstrate whether/when a stable endpoint is reached. |
| Independent dose-response replication and broader concentration range | Quantify experimental variability and strengthen isotherm-model parameter uncertainty and model comparison. |
| Leaching under water contact, runoff, and wet–dry cycling | Quantify chromium and soluble-component release from binder, mortar, and future full mixtures. |
| Chromium speciation before and after adsorption/incorporation | Differentiate Cr(VI), Cr(III), total chromium, and possible redox transformations. |
| Real tannery wastewater testing | Assess effects of pH, salts, sulfides, organic matter, suspended solids, oils, dyes, and competing ions. |
| Surface area, mineral phases, pH_pzc, zeta potential, and surface functional groups | Support any future mechanistic interpretation of pH-dependent uptake and interactions involving hydroxyapatite-rich and carbonaceous domains. |
| Direct residue characterization (XRD, FTIR, SEM–EDS/ICP, and where appropriate XPS) | Identify mineral/chemical phases and distinguish BC-derived material, acid/salt residues, and chromium-bearing components after drying. |
| Total chromium and Cr(VI)/Cr(III) analysis of the residue | Close the chromium mass balance and separate aqueous Cr(VI) depletion from chromium retention and possible redox transformation. |
| Regeneration efficiency and adsorbent stability across repeated adsorption–desorption cycles | Evaluate cycle-to-cycle performance, material stability, and the chromium-bearing regenerant stream if regeneration is pursued. |
| Independent binder and mortar preparation replicates | Support formal statistical inference rather than descriptive screening of subsamples and repeated measurements. |
| Storage stability and workability assessment | Evaluate dispersion stability and practical handling of the complete process-derived residue in asphalt binder. |
| Full asphalt mixture design with verified volumetrics | Move from binder/mortar compatibility to pavement-relevant material performance with measured air voids and bulk properties. |
| Aging, moisture, abrasion, cracking, and end-of-life recycling scenarios | Evaluate long-term performance and environmental exposure conditions relevant to asphalt matrices. |
